# Elucidation of Potential Genotoxicity of MXenes Using a DNA Comet Assay

**DOI:** 10.1021/acsabm.4c01142

**Published:** 2024-12-03

**Authors:** Sergiy Kyrylenko, Inna Chorna, Zhanna Klishchova, Ilya Yanko, Anton Roshchupkin, Volodymyr Deineka, Kateryna Diedkova, Anastasia Konieva, Oksana Petrichenko, Irina Kube-Golovin, Gunther Wennemuth, Emerson Coy, Iryna Roslyk, Ivan Baginskiy, Veronika Zahorodna, Oleksiy Gogotsi, Benjamin Chacon, Luciana P. Cartarozzi, Alexandre L. R. Oliveira, Igor Iatsunskyi, Yury Gogotsi, Maksym Pogorielov

**Affiliations:** † Biomedical Research Center, 187506 Sumy State University, 31 Sanatorna Street, Sumy 40007, Ukraine; ‡ 61769 Federal University of Lavras UFLA, Lavras, Minas Gerais CEP 37203-202, Brazil; § University of Latvia, Institute of Atomic Physics and Spectroscopy, 3 Jelgavas Street, Riga LV-1004, Latvia; ∥ Department of Anatomy, University Hospital, University Duisburg-Essen, Hufelandstr. 55, Essen D-45147, Germany; ⊥ Materials Research Centre, 3 Krzhizhanovskogo Street, Kyiv 03680, Ukraine; # A.J. Drexel Nanomaterials Institute and Departmental of Materials Science and Engineering, 6527 Drexel University, 3141 Chestnut Street, Philadelphia, Pennsylvania 19104, United States; ¶ NanoBioMedical Centre, 529746 Adam Mickiewicz University, 3, Wszechnicy Piastowskiej Str., Poznan 61-614, Poland; ∇ Laboratory of Nerve Regeneration, Institute of Biology, 28132 University of Campinas, Campinas, São Paulo 13083-862, Brazil

**Keywords:** Ti_3_C_2_T_*x*_, Nb_4_C_3_T_*x*_, MXene, DNA comet assay, DNA fragmentation, cell viability, resazurin reduction assay, cell death, electrophoresis

## Abstract

MXenes are among the most diverse and prominent 2D materials. They are being explored in almost every field of science and technology, including biomedicine. In particular, they are being investigated for photothermal therapy, drug delivery, medical imaging, biosensing, tissue engineering, blood dialysis, and antibacterial coatings. Despite their proven biocompatibility and low cytotoxicity, their genotoxicity has not been addressed. To investigate whether MXenes interfere with DNA integrity in cultured cells, we loaded the cells with MXenes and examined the fragmentation of their chromosomal DNA by a DNA comet assay. The presence of both Ti_3_C_2_T_*x*_ and Nb_4_C_3_T_*x*_ MXenes generated DNA comets, suggesting a strong genotoxic effect in murine melanoma and human fibroblast cells. However, no corresponding cytotoxicity was observed, confirming that MXenes were well tolerated by the cells. The lateral size of the MXene flakes was critical for developing the DNA comets; submicrometer flakes induced the DNA comets, while larger flakes did not. MXenes did not induce DNA comets in dead cells. Moreover, the extraction of the chromosomal DNA from the MXene-loaded cells or mixing the purified DNA with MXenes showed no signs of DNA fragmentation. Unconstrained living MXene-loaded cells did not show cleavage of the DNA with MXenes under electrophoresis conditions. Thus, the DNA comet assay showed the ability of submicrometer MXene particles to penetrate living cells and induce DNA fragmentation under the applied field. The most probable mechanism of DNA comet formation is the rotation and movement of submicrometer MXene flakes inside cells in an electric field, leading to cleavage and DNA shredding by MXene’s razor-sharp edges. Under all other conditions of interest, titanium- and niobium-carbide-based MXenes showed excellent biocompatibility and no signs of cytotoxicity or genotoxicity. These findings may contribute to the development of strategies for cancer therapy.

## Graphical abstract

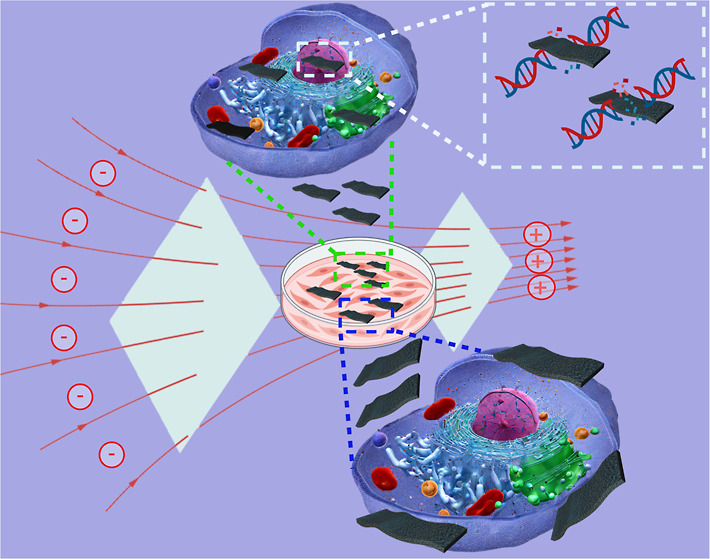

## 1 Introduction

Biocompatibility, the ability to fulfill an intended medical function without causing local or systemic adverse effects, is a primary consideration when evaluating new biomaterials. It encompasses the absence of cytotoxicity, genotoxicity, immunotoxicity, or tissue irritation. Only after a comprehensive assessment of these properties can we investigate the effectiveness of biomaterials, including in vivo testing and clinical trials. Several factors can influence the biocompatibility performance of nanomaterials, including chemical composition, size, shape, degradation characteristics, ion release capability, etc. These factors should be thoroughly explored during toxicological experiments.

The isolation of single-layer graphene in 2004 has motivated the search for new two-dimensional (2D) materials.^1^ They include transition metal dichalcogenides, hexagonal boron nitride, graphitic carbon nitride, and black phosphorus.^2–4^ Highly promising hydrophilic 2D materials with high conductivity and a wide range of possible applications, MXenes, were discovered at Drexel University in 2011. With the general formula M_*n*+1_X_*n*_T_*x*_, where *M* is an early transition metal, *X*–carbon or nitrogen, and *T*_*x*_–the surface terminations, such as O, OH, F, and/or Cl, they are represented by more than 40 already synthesized stoichiometric compositions and dozens of solid solutions. Many more have been explored by computational methods.^5^ MXenes have demonstrated significant potential for applications in very diverse fields, including lithium and sodium-ion batteries, electrocatalysis, optoelectronic devices, flexible electronics, and healthcare, including cancer treatment, bacteriology, immunology, targeted drug delivery, tissue engineering, etc.^6–10^ Although MXenes are still a new subject in biomedical research, it is becoming evident that MXenes will soon find wide use in various applications and, hence, will come into close contact with human bodies, tissues, and cells. Investigating their biocompatibility is essential to the intensive exploration of MXenes for biomedical applications. Assessment of the biosafety of MXenes is also crucial regarding the environmental consequences of their widespread use.

The most widely used MXenes in biomedical research are titanium and niobium carbides, which have already demonstrated their potential for cancer diagnostics and treatment as well as in tissue engineering. Pure MXenes and their modifications are used with a wide range of flake sizes and various *T*_*x*_ terminations.^11–15^ While both Ti- and Nb-based MXenes have shown a high degree of biocompatibility, it is noteworthy that conflicting information regarding safety levels exists, even within the same category of 2D materials. The study of A.M. Jastrzębska revealed that Ti_3_C_2_T_*x*_ MXenes exhibited no cytotoxicity toward HaCaT and human alveolar basal epithelial cells (A549) at concentrations up to 500 mg/L. However, the viability of cancer cells (MRC-5 and A375) declined at MXene concentrations exceeding 250 mg/L.^16^ Later, they demonstrated similar results with Ti_2_NT_*x*_ MXenes using MCF-7, A365, MCF-10 A, and HaCaT cells.^17^ Other findings revealed a similar toxicity profile of Ti_3_C_2_T_*x*_ for human umbilical vein endothelial cells (HUVECs) after 48 h of cocultivation.^18^ On the other hand, experiments with primary neural stem cells, NSCs-derived differentiated cells,^19^ and human mesenchymal stem cells (hMSCs)^20^ demonstrated a decrease in cell viability at a concentration above 20 mg/L. In addition, M. Gu showed that Ti_3_C_2_T_*x*_ quantum dots exhibit high toxicity at concentrations above 50 mg/L.^21^ Finally, the study of A. Rozmysłowska-Wojciechowska determined that Ti_3_C_2_T_*x*_ MXene at concentrations exceeding 62.5 mg/L led to a reduction in the viability in various cell lines, including human skin malignant melanoma (A375), human immortalized keratinocytes (HaCaT), human breast cancer (MCF-7), and mammary epithelial cells (MCF-10A).^22^ Interestingly, when these cells were incubated in the presence of collagen-modified MXenes, a statistically significant increase in viability was observed across all of the cell cultures examined. Overall, the surface modifications of MXenes with both organic and inorganic substances enhance their biocompatibility. However, Ti_3_C_2_T_*x*_-SP (soybean protein),^23^ MnO_*x*_/Ti_3_C_2_T_*x*_-SP^24^, Ti_3_C_2_T_*x*_-IONPs-SP^25^, and Ti_3_C_2_T_*x*_-PEG (polyethylene glycol)^26^ showed no apparent cytotoxicity when tested on breast 4T1 cancer cells. These tests were conducted over a concentration range of 10–400 mg/L during 48 h cocultivation. No toxicity of MXene@PVA hydrogel, SF@MXene biocomposite film, and PCL-MXene electrospun membranes in NIH-3T3,^27^ fibroblast-HSAS1,^28^ and primary fibroblast^15^ cultures. On the other hand, J. Zhang demonstrated that Ti_3_C_2_T_*x*_–PVP composite significantly decreased the proliferation of bone marrow-derived mesenchymal stem cells (BMSCs) at concentrations above 50 mg/L^29^.

From the data available, it is evident that the biocompatibility of Nb-based MXenes surpasses that of their Ti counterparts. Various Nb-based materials, including Nb_2_C QDs,^21^ Nb_2_C-MSNs-SNO,^30^ Nb_2_C-PVP,^31^ and the CTAC@Nb_2_C-MSN-PEG^32^ composite displayed remarkable biocompatibility with HUVEC, 4T1, and glioma U87 cancer cells, even at concentrations up to 200 mg/L. Notably, M. Gu’s findings indicated that Nb_2_CT_*x*_ exhibited significantly higher cellular viability in the 50 to 100 mg/L concentration range than Ti_3_C_2_T_*x*_.^21^

Genotoxicity refers to the ability of a substance to cause damage to genetic information within a cell. This damage can be manifested as mutations, chromosomal aberrations, or DNA strand breaks. Genotoxic substances have the potential to lead to detrimental effects, such as cancer, birth defects, and other genetic disorders. Therefore, genotoxicity assessment is paramount to estimating the safety of various nanomaterials. Various experimental assays, including in vitro and in vivo studies, are used to assess the genotoxic potential of substances. These include the Ames test, chromosomal aberration assay, micronucleus assay, sister chromatid exchange assay, etc. Among these, the DNA comet assay (single-cell gel electrophoresis) can directly detect DNA fragmentation in individual cells, including strand breaks and alkali-labile sites. It is based on the migration of the fragmented DNA in an electric field, forming a comet-like tail, which provides a visual indicator of DNA damage.^33,34^

It was indicated that certain types of nanomaterials may exhibit genotoxic effects. The genotoxic potential of nanomaterials is influenced by various factors, including their chemical composition, size, shape, surface chemistry, and specific biological systems in which they are tested. In particular, it was shown that graphene and graphene-based materials have the potential to induce genetic damage. However, the type of damage depends on the material.^35^ The genotoxicity of other 2D nanomaterials has been studied less. It was demonstrated that neither mechanically exfoliated nor chemical vapor deposition–grown transition metal dichalcogenides affect cellular viability or induce genetic defects in the in vitro model of human epithelial kidney cells.^36^ Hexagonal boron nitride and other 2D nanomaterials were also shown to be neither cytotoxic nor genotoxic in the human gastrointestinal epithelium triculture model in vitro.^37^ Similarly, graphitic carbon nitride was not genotoxic and even protective from cadmium and arsenic genotoxicity in plants.^38^ The genotoxic properties of black phosphorus still require further investigations, although it is known to have excellent biocompatibility.^39^ Not only nanomaterials but also the products of their metabolization in biological systems should be evaluated for their genotoxicity. The end product of the metabolization of Ti_3_C_2_T_*x*_ MXene could be titanium dioxide (TiO_2_). The genotoxic profile of TiO_2_ nanoparticles remains to be concluded.^40^ However, it was reported that the cells loaded with the TiO_2_ nanoparticles showed positive results in the DNA comet assay while showing no changes in cytotoxicity markers, such as lipid peroxidation, ROS formation, or changes in the composition of cell membranes. It was deduced that the comets observed in the cells with TiO_2_ nanoparticles resulted from the false-positive DNA comet assay.^41^

To date, the genotoxic properties of MXenes have yet to be addressed. Therefore, the aim of this study was to investigate the genotoxicity of MXenes, particularly their ability to induce fragmentation of the chromosomal DNA in cultured cells using the DNA comet assay.

## 2 Materials and Methods

### 2.1 Preparation and Characterization of MXenes

MXene synthesis and characterization. To synthesize MXenes, aluminum was etched from their MAX phase precursors, Ti_3_AlC_2_ and Nb_4_AlC_3_. Ti_3_AlC_2_ was purchased from Carbon Ukraine, and Nb_4_AlC_3_ was produced at Drexel University by mixing a 4:1.1:2.7 atomic ratio of Nb/Al/C. The powder mixture was then mixed with 5 mm alumina balls in a 2:1 ball/powder ratio. This mixture was ball milled at 60 rpm for 24 h before high-temperature annealing at 1650 °C for 4 h. MXenes were synthesized by the wet chemical etching of the MAX phase. For Ti_3_C_2_T_*x*_, the MAX phase was etched with a 2:3 ratio of 50% HF: 12 M HCl at 35 °C for 24 h. For Nb_4_C_3_T_*x*_, the MAX phase was etched with 50% HF at 50 °C for 7 days. The etched multilayer MXenes were then delaminated with LiCl for Ti_3_C_2_T_*x*_ or TMAOH for Nb_4_C_3_T_*x*_ by stirring overnight at room temperature. The mixtures were then centrifuged until they reached neutral pH and collected via centrifugation. The concentration of MXenes was measured by (a) spectrophotometry, measuring absorbance at certain wavelengths, and (b) measuring the dry weight of the colloid pellet after evaporation of the solvent from a specific volume.

To reduce flake size, delaminated MXene colloidal solutions were probe sonicated (Sonic Dismembrator, Fisher FB505, 500 W, USA) under pulse setting (8 s on pulse and 2 s off pulse) at an amplitude of 50% in an ice bath. The size distributions of the 2D MXene sheets were obtained by using dynamic light scattering (DLS). The samples were diluted to 10 μg/mL in DI water. DLS analysis was conducted using a Malvern Zetasizer Nano ZS (Malvern Instruments, UK) equipped with a backscattered light detector operating at a 173° angle. Each sample went through 3 runs involving 12 averaged scans. The runs were averaged to yield the final average MXene diameter (Supporting information Figure S1) with corresponding zeta potential (Supporting Information Table ST1). UV–vis spectra were collected from 300 to 1000 nm using Thermo Scientific, Evolution 201 (Supporting Information Figure S2).

The produced MXenes were characterized by X-ray diffraction (XRD), which showed the shifting of the 002 peaks to a lower 2θ angle, indicating an increase in interlayer spacing upon removal of Al during etching. The XRD analyses were performed on the MAX phase powder and vacuum-filtered films of delaminated MXene (Supporting Information Figure S3). The morphology and chemical composition of Ti_3_C_2_T_*x*_ and Nb_4_C_3_T_*x*_ were analyzed by scanning electron microscopy (SEM) using a JEOL JSM7001F. Transmission electron microscopy (TEM) was done using a JEOL ARM 200F operating at 200 kV and equipped with an EDX analyzer. Additionally, a Renishaw micro-Raman spectrometer was utilized to examine these properties further.

### 2.2 Treatment of Cells with MXenes

B16F10 mouse melanoma cells and primary human dermal fibroblasts (DFB, passage 8) were cultivated in DMEM/F12 medium with 10% FBS and antibiotics/antimycotics (all sourced from Thermo Fisher Scientific) using standard conditions.^11^ For comet assays, the cells were plated into 6-well plates at 10,000 cells/cm^2^ in a 2 mL medium. The next day, MXenes were added to final concentrations and kept in contact with the cells for 4 h or otherwise as indicated (Supporting Information Figure S4). After that, the MXenes were washed away with PBS (Supporting Information Figure S5), and the cells were further grown under standard conditions as indicated. To investigate the influence of serum on the interaction of the MXenes with the cells, working dilutions of the Ti_3_C_2_T_*x*_ MXene were made in the complete medium and the serum-free medium (Supporting Information Figure S6) and added to the cells for 4 h, after which the MXene was washed out. The cells were further cultivated under normal conditions as indicated. For the DNA comet assays, the cells were taken by trypsinization and counted. The cell counts were somewhat lower in the wells treated with MXene (Supporting Information Table ST2), although the treated cells showed no visible signs of MXene cytotoxicity (Supporting Information Figure S7).

### 2.3 DNA Comet Assay

Fragmentation of chromosomal DNA in living cells was evaluated by the DNA comet assay under alkaline conditions as described^42^ and quantified previously.^43^ Suspension of cells (2 × 10^5^ cells in 200 μL) was mixed with an equal volume of 1% low-melting-point agarose (Sigma-Aldrich) in PBS kept in liquid at 37 °C. 75 μL portion of this mixture was spread on the microscope glass slides precoated with 1% agarose (Sigma-Aldrich) and dried on air. The cell suspension on the glass slide was quickly covered with a coverslip and placed on ice for 5 min to solidify the agarose. As a positive control, cells treated with 10 μM H_2_O_2_ for 3 min at 4 °C were used (H_2_O_2_ causes damage to DNA by generating hydroxyl free radicals). After removal of the coverslips, the slides were immersed in cold lysis solution (2.5 M NaCl, 100 mM EDTA, 10 mM Tris base, and 1% Triton X-100, pH 10) and kept for 1 h at 4 °C in the dark. After lysis, the slides were rinsed with cold distilled water and placed side by side, avoiding spaces between them, in a horizontal electrophoresis chamber filled with a cold alkaline electrophoresis running buffer (300 mM NaOH and 1 mM EDTA, pH 13). After 20 min of soaking in the alkaline running buffer, electrophoresis was performed at a 0.8 V/cm voltage for 20 min. Then, the slides were neutralized in a neutralization buffer (0.4 M Tris–HCl pH 7.4) for 10 min and rinsed twice with cold distilled water for 5 min. Slides were dried, stained with DAPI, and analyzed with a fluorescence microscope. 100 comets per slide were assessed using the Open Comet software.^44^ For the time course experiments, the longest time points (72 h) were treated first, while the wells with the cells for the shorter time points were still growing under normal conditions in parallel wells and treated consecutively such that the cells were taken for the DNA comet assay at the same time. For control experiments with metabolically inactivated cells, the cells were killed by heat or ethanol. The cells were collected by trypsinization for heat-induced killing and incubated at 60 °C for 30 min in a water bath.^45^ For ethanol-induced killing,^46^ the 6-well plate was washed with PBS and incubated in 2 mL of 20% ethanol in PBS for 30 min. The detached cells were collected by centrifugation. The MXene was added to the cells, incubated as indicated, and proceeded with the DNA comet assay protocol. Metabolic inactivation of the cells was confirmed by a resazurin reduction assay.

### 2.4 Quantification of DNA Comets

The DNA comet assay results were calculated by determining the tail moment (TM) and olive tail moment (OTM) parameters using Open Comet v1.3.1 software. This software measures the length of the comet tails, which corresponds to the relative mobility of the DNA fragments, and the pixel intensity of the tails as the percentage of DNA distribution in the tails versus the heads (nuclei). These parameters correspond to the intensity of the DNA fragmentation and hence the damage to the DNA in the cell. The software was set to recognize the images automatically, adapting to images with various magnifications and including adjustments to the background noise. An example of image processing is shown in Supporting Information Figure S8. Both TM and OTM parameters reflect the extent of the DNA damage. However, we presume that the OTM parameter can be regarded as more informative as it takes into account the amount of DNA in the comet heads more accurately (eqs 1 and 2); therefore, we represented the results of the DNA comet assay as OTM.

1 $$TM=\frac{tail\;length\times tail\;\%\;DNA}{100}$$

2 $$OTM=\frac{tailmean-headmean}{100}\times tail\;\%\;DNA$$

Statistical significance was estimated using GraphPad Prism v9.5 using ordinary one-way ANOVA with Dunnett’s multiple comparisons test with statistical significance (*p*-values) indicated by asterisks, where * means *p* ≤ 0.05, ** *p* ≤ 0.01, *** *p* ≤ 0.001, and **** *p* ≤ 0.0001.

### 2.5 Assessment of Apoptosis

For morphological visualization of the apoptotic bodies in the same cell populations, which were used for the comet assays, aliquots of the cells were fixed in methanol-acetic acid (3:1), spread onto the glass slides, and observed under a fluorescence microscope after DAPI staining (1 μg/mL in 100 mM NaCl, 10 mM EDTA, and 10 mM Tris–HCl, pH 7.4).

The possibility of the induction of apoptosis or necrosis by Ti_3_C_2_T_*x*_ and Nb_4_C_3_T_*x*_ MXenes was further assessed by flow cytometry using annexin V and propidium iodide (PI). B16F10 cells were incubated with Ti_3_C_2_T_*x*_ and Nb_4_C_3_T_*x*_ MXenes at 100 μg/mL for 24 h. After incubation, the cells were washed with ice–cold DMEM and incubated with 0.25 μg/mL annexin V (Immunotools, Germany)/DMEM for 20 min at 4 °C. Then, the cells were washed with ice–cold DMEM and stained with 1 μg/mL PI/DMEM. The stained cell samples were examined in a FACScalibur flow cytometer (Becton Dickinson, USA) and analyzed by CellQuest Pro Version 6 (Becton Dickinson, USA).

### 2.6 Assessment of the Metabolic Activity of Cells

The resazurin reduction assay was used to monitor the relative number of cells (their metabolic activity) as described.^11^

### 2.7 Transmission Electron Microscopy

B16F10 melanoma cells were plated at 15,000 cells per cm^2^ on ACLAR fluoropolymer inserts in 24-well plates. After 24 h in culture, 180 and 3000 nm Ti_3_C_2_T_*x*_ MXene fractions were added at 25 μg/mL, and the cells were maintained for a further 24 h. After this, the culture medium was removed, and the cells were fixed with Karnovsky’s solution (2.5% glutaraldehyde and 1% paraformaldehyde in sodium phosphate buffer −0.1 M, pH 7.38) for 20 min. The samples were then washed in buffered cacodylate (0.15 M, pH 7.38) and postfixed in 1% reduced osmium tetroxide (3% potassium ferrocyanide in 0.15 M cacodylate buffer plus 4 mM calcium chloride) for 15 min, followed by incubation with thiocarbohydrazide (TCH) for 5 min. After washing in water (3 times for 5 min), a second osmium tetroxide fixation was applied for 15 min, followed by further washing in water (3 × 5 min) and overnight incubation in 1% uranyl acetate (in water). The samples were then washed in water, incubated in lead citrate for 10 min, and dehydrated using increasing concentrations of ethyl alcohol (30%, 50%, 70%, 90%, and 100%, 10 min each) and acetone (15 min). Finally, the fluoropolymer inserts with adherent cells were placed into individual silicone embedding molds and embedded in resin (Durcupan, Fluka). Polymerization of the resin was carried out in an incubator at 60 °C for 5 days.

After resin polymerization, the blocks were trimmed, and semithin sections (0.5 μm thick) were cut using a Leica Ultracut UC-7 ultramicrotome. The semithin sections were stained with 0.25% toluidine blue and examined under a light microscope to check the availability of cells and the quality of the sections. The blocks were then further trimmed, and ultrathin sections (70 nm thick) were cut with the same ultramicrotome, collected on Formvar-coated single-slot grids, and observed and documented using a transmission electron microscope (FEI Tecnai G2 Spirit BioTwin) operating at 80 kV.

### 2.8 DNA Extraction and Analysis

To verify the integrity of the chromosomal DNA in cells developing comets, the cells were prepared the same way as for the DNA comet assay running in parallel. Then, instead of spreading the cells onto the glass slide and subjecting them to the DNA comet assay, the cells were lyzed, and the chromosomal DNA was extracted using conventional methods. This way, the cells treated with Ti_3_C_2_T_*x*_ MXene in parallel with the cells for the DNA comet assay were collected by trypsinization and spun down the same way as for the DNA comet assay. The DNA comet assay was run in parallel to verify the effect of MXene’s presence on the DNA comets’ appearance. The cells from one well of the 6-well plate were resuspended in 200 μL PBS and lyzed by adding SDS to 1% and Proteinase K (Sigma-Aldrich) to 100 μg/mL. The mixture was kept at 56 °C for 30 min, after which the DNA was purified with phenol saturated against 0.1 M Tris–HCL pH 7.5, then with a phenol/chloroform mixture, and then the traces of phenol were removed by additional chloroform extraction. The DNA was precipitated using ethanol, rinsed with 70% ethanol, dried on air, and dissolved in 100 μL of TE buffer (10 mM Tris–HCl pH 7.5, 1 mM EDTA). The concentration and purity of the obtained DNA were estimated by a μDrop Duo Plates in Multiskan SkyHigh microplate reader (Thermo Fisher Scientific) by measuring absorbances at 260 and 280 nm. The obtained DNA was free from MXenes as phenol extraction effectively removes MXenes (Supporting Information Figure S9).

The DNA was analyzed in 0.8% agarose gel electrophoresis in buffer 0.5× TBE (44.5 mM Tris-borate, 1 mM EDTA) at 8 V/cm for 45 min or otherwise, as indicated. The gel was stained with ethidium bromide (EtBr) and photographed with a UV transilluminator.

To check the possibility that MXenes can cut the chromosomal DNA under gel electrophoresis conditions, we modulated the conditions of the DNA comet assay during electrophoresis in an agarose gel. For this, 1 μg of the purified intact chromosomal DNA from mouse melanoma cells was mixed with various quantities of Ti_3_C_2_T_*x*_ MXene (ratios DNA/MXene from 1:50 to 1:1600). The mixture of the DNA with MXenes was embedded into low-melting-point agarose as for the DNA comet assay. Then, the DNA/MXene in still-not-solidified agarose was loaded into the wells of the 0.8% agarose gel in 0.5× TBE, and the electrophoresis was run and analyzed as usual.

In another control experiment, agarose gel with fragments filled with agarose mixed with MXene was prepared. For this, the fragments of the gel 6 × 10 mm were cut in front of the wells out of the 0.8% agarose gel in 0.5× TBE, and the space was filled with the agarose of the same concentration and composition but supplied with various quantities of Ti_3_C_2_T_*x*_ MXene from 0.07 mg/mL up to 2.2 mg/mL with 2× increment (Supporting Information Figure S10). The intact chromosomal DNA from mouse melanoma cells (1.25 μg per lane) was loaded into the wells, and electrophoresis was run as usual at 8 V/cm for 45 min, followed by EtBr staining and UV visualization.

### 2.9 In Vitro Electrophoresis

Electrophoretic inserts for 6-well plates were 3D printed out of polylactic acid, as shown in Supporting Information Figure S11. Each insert was supplied with 2 platinum wires as electrodes (⌀ 0.75 mm, 90% Pt, and 10% *R*_h_) such that 2 cm of the wires were in contact with the bottom of the well in pairs opposite each other. The B16F10 mouse melanoma cells were plated into 6 well plates at 10,000 cells per cm^2^ in 2 mL of the complete cell culture medium. The next day, Ti_3_C_2_T_*x*_ or Nb_4_C_3_T_*x*_ MXene were added to the cells at concentrations as indicated. After 24 h, 2 mL of more medium was added to the wells. The inserts with platinum electrodes were carefully inserted into the wells, and the wires were connected to the electrophoresis power supply. The electric field was applied at 4 V (∼1.3 V/cm), and the electrophoresis was run for 10 min or as indicated otherwise (the current was ∼4 mA when one insert in one well was connected). For each 5 min, the current was paused, and the medium was gently mixed by shaking to equalize the changes in the pH due to the electric current. The temperature in the wells during the in vitro electrophoresis was monitored by the hand-held infrared thermometer, while no substantial changes were recorded (not shown). Immediately after the electrophoresis, resazurin was added to the medium to 15 μg/mL and incubated overnight or otherwise as indicated. The viability of the cells was calculated as described above.

To verify if MXene can cause DNA fragmentation in the electric field under conditions similar to those in the electrophoresis stage during the DNA comet assay but in living cells, we performed extraction of chromosomal DNA from the cells immediately after in vitro electrophoresis. For this, in vitro electrophoresis was done as described above, the medium was removed, and the cells were lysed directly in the wells by adding 400 μL of lysis buffer containing 1% SDS and 100 μg/mL of Proteinase K in PBS. The lysates were transferred to the 1.5 mL Eppendorf tubes and incubated at 56 °C for 30 min. After that, the DNA was purified with phenol/chloroform, and ethanol precipitated as described above. The integrity of the DNA was estimated through gel electrophoresis in 0.8% agarose in 0.5× TBE, as described above.

To eliminate the influence of the electric current with concomitant changes in pH, we placed the whole cell culture plate with the MXene-loaded cells between electrodes 30 cm apart, at which 20,000 V potential was applied for 20 min, after which cell viability was assessed by resazurin reduction assay.

## 3 Results

### 3.1 MXene Characterization

To reveal the structural and compositional features of the produced MXenes, we used SEM and TEM, as well as Raman spectroscopy. The SEM analysis revealed that the freshly prepared Ti_3_C_2_T_*x*_ MXene had an average lateral size of 4.5 ± 1 μm, while Nb_4_C_3_T_*x*_ MXene exhibited a smaller average size of 295 ± 85 nm (Figure 1A, Supporting Information Figure S12). Upon application of ultrasonication for size reduction, Ti_3_C_2_T_*x*_ flakes were fractionated into distinct size ranges: Fr180 at 290 ± 120 nm, Fr600 at 640 ± 40 nm, Fr1000 at 1220 ± 310 nm, and Fr3000 at 2620 ± 450 nm (Supporting Information Figure S12). These results correlated well with the DLS analysis and indicated successful size reduction and fractionation into specific nanoscale dimensions. Furthermore, the SEM images confirmed that the MXene samples contained single- and few-layer flakes.

**Figure 1:**
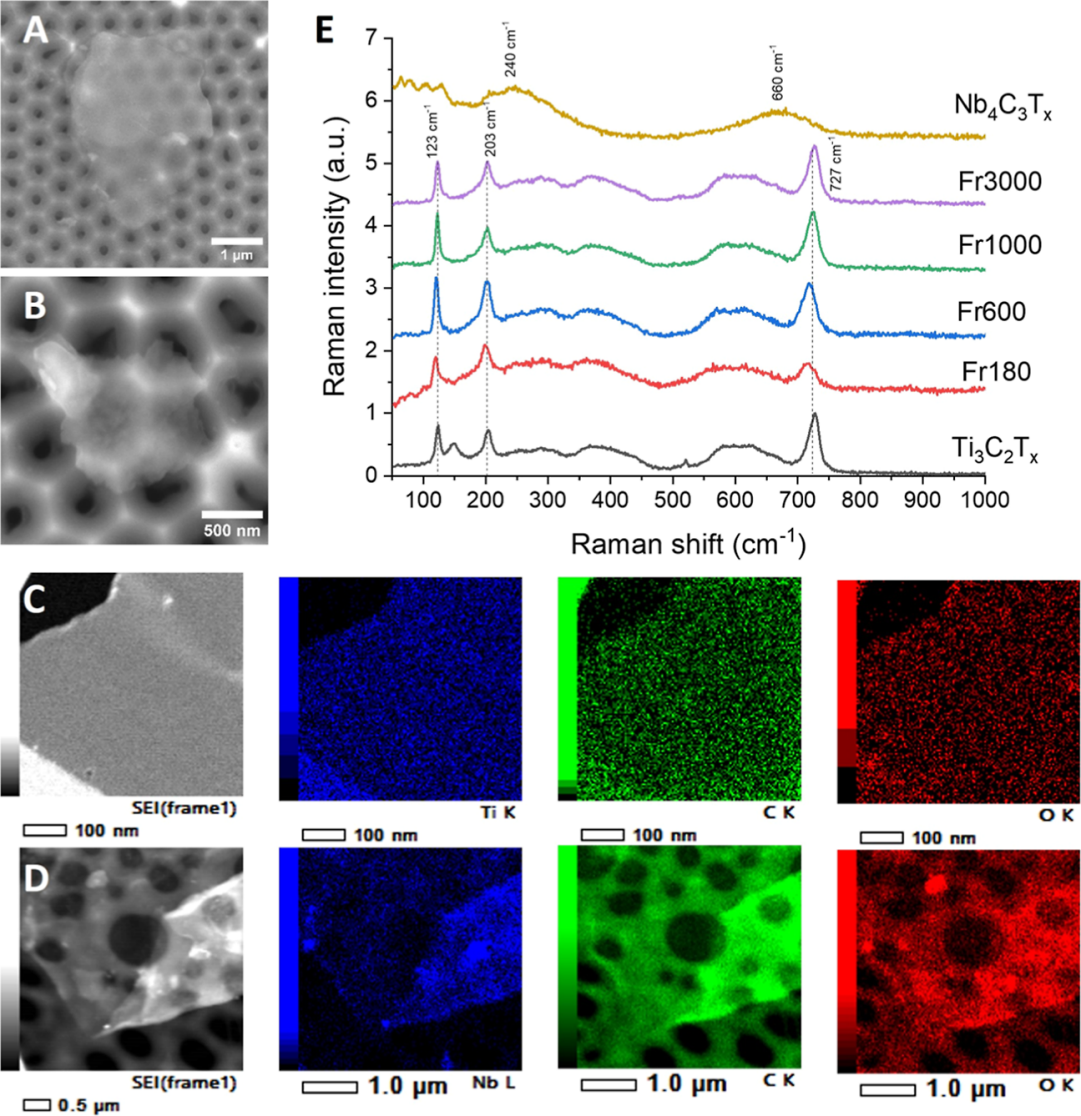
SEM images of (A) Ti_3_C_2_T_*x*_ and (B) Nb_4_C_3_T_*x*_ MXenes; TEM analysis with EDX mapping of (C) Ti_3_C_2_T_*x*_ and (D) Nb_4_C_3_T_*x*_ MXenes; (E) Raman spectra of MXene samples.

We then conducted TEM analysis with EDX (Figure 1C,D, Supporting Information Figure S12). The TEM analysis of pristine Ti_3_C_2_T_*x*_ and Nb_4_C_3_T_*x*_ revealed few-layer flakes, with some monolayer MXene being used for titanium carbide. EDX mapping, presented in an accompanying image, confirmed the composition of Ti_3_C_2_T_*x*_ as primarily titanium, carbon, and oxygen, while Nb_4_C_3_T_*x*_ contained niobium, carbon, and oxygen. The reduced-size MXenes were also included (Supporting Information Figure S12), showing lateral sizes that correlated well with EDX and DLS measurements. In some instances, the MXene flakes were observed to have small inclusions averaging less than 100 nm, which could be small pieces of MXene.

Finally, Raman spectroscopy (Figure 1E) was employed to verify the structure and phase of MXene before and after size reduction. It is well established that Ti_3_C_2_T_*x*_ MXene exhibits several characteristic features within the 100–800 cm^–1^ range.^47,48^ Specifically, sharp Raman peaks are observed at 123 (*E*_g_), 203 (*A*_1g_), and 727 (*A*_1g_(C)) cm^–1^, indicating delaminated Ti_3_C_2_T_*x*_ sheets. The quality of the Ti_3_C_2_T_*x*_ flakes can be assessed by the ratios of the Raman peak intensities *I*_204_/*I*_727_ and *I*_204_/*I*_123_. For the pristine Ti_3_C_2_T_*x*_ flakes, these values were determined to be *I*_204_/*I*_727_ = 0.86 and *I*_204_/*I*_123_ = 0.95. After the size reduction, we obtained values of *I*_204_/*I*_727_ = 1.11 and *I*_204_/*I*_123_ = 1, *I*_204_/*I*_727_ = 1 and *I*_204_/*I*_123_ = 0.96, *I*_204_/*I*_727_ = 0.85 and *I*_204_/*I*_123_ = 0.85, and *I*_204_/*I*_727_ = 0.84 and *I*_204_/*I*_123_ = 1, for Fr180, Fr600, Fr1000, and Fr3000 samples, respectively. As demonstrated in the previous studies, those values indicate the presence of a few-layer Ti_3_C_2_T_*x*_ MXene.^47,48^ Raman spectroscopy was also employed to investigate Nb_4_C_3_T_*x*_, indicating two broad peaks at around 240 and 660 cm^–1^ corresponding to Nb–O and Nb–C vibrational modes, respectively.^49^

### 3.2 DNA Comets in Live Cells in the Presence of MXene

Cells were treated with various concentrations of Ti_3_C_2_T_*x*_ MXene (6.25, 25, and 100 μg/mL) for 4 h and incubated for 2 days, after which the cells were extracted by trypsinization and subjected to the alkaline DNA comet assay. Figure 2 shows that the cells developed visible DNA comets in the presence of MXene. All concentrations of MXene tested produced DNA comets. We investigated the possible effect of serum present in the complete cell culture medium on the ability of MXene to induce comets. For this, we treated the cells with MXene for 4 h in a serum-free medium and cultivated them for 2 days in the complete medium under normal conditions. We found that incubation of the cells with MXene in both the complete medium and in serum-free medium for 4 h did not have any consistent and substantial effect on the appearance and intensity of the DNA comets (Figure 2A, lower left image). The intensity of the comets generally increased with the concentration of MXene. However, at lower concentrations, the effect of the concentration was weaker and less consistent (Figure 2B). Treatment for 3 min with H_2_O_2_, the commonly used control inducing genotoxicity (final H_2_O_2_ concentration in cells was 10 μM), resulted in clearly visible DNA comets. However, the intensity of the comets was lower than that in the case of the highest concentration of MXene (100 μg/mL). We concluded that Ti_3_C_2_T_*x*_ MXene in live mouse melanoma cells induced a robust appearance of the DNA comets under standard conditions of the alkaline DNA comet assay, which might suggest a genotoxic effect of the MXene on cultured cells in vitro.

**Figure 2:**
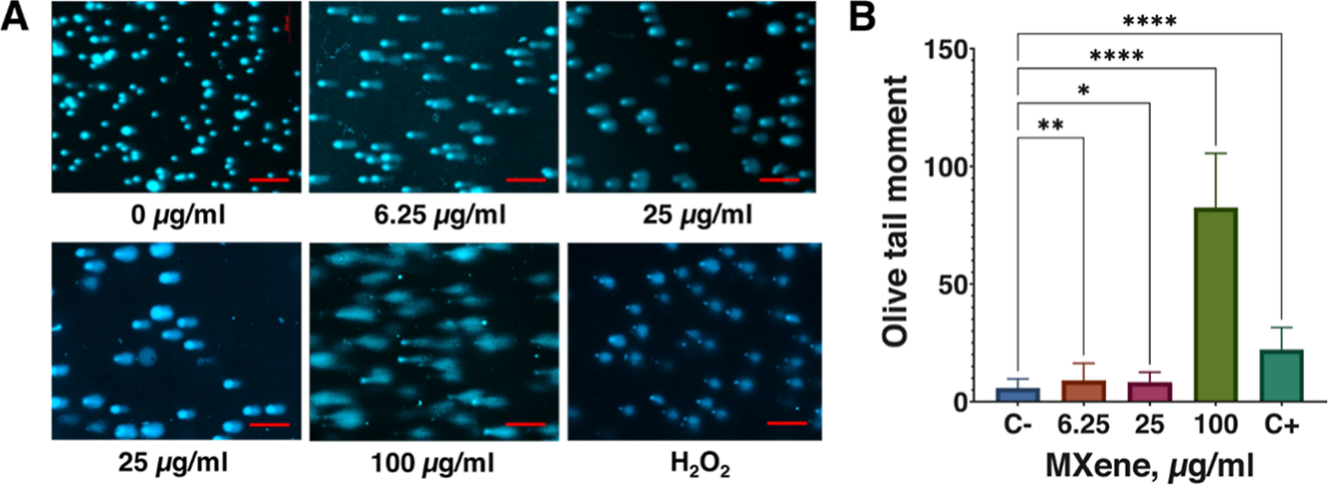
DNA comet assay in B16F10 mouse melanoma cells loaded with Ti_3_C_2_T_*x*_ MXene. (A) cells treated with various concentrations of MXene and subjected to the DNA comet assay under alkaline conditions, scale bars = 200 μm. (B) results quantified as the OTM of the comets, where C- represents negative control (untreated cells) and C+ represents positive control (cells treated with 10 μM H_2_O_2_).

### 3.3 Ti_3_C_2_T_*x*_ and Nb_4_C_3_T_*x*_ Induced DNA Comets in Mouse Melanoma and Human Fibroblast Cells

We addressed the question of whether the observed induction of the DNA comets was specific to Ti_3_C_2_T_*x*_ MXene and mouse melanoma cells. First, we applied Ti_3_C_2_T_*x*_ to normal human fibroblasts in a culture. We found that Ti_3_C_2_T_*x*_ MXene could induce DNA comets in fibroblasts similarly to the mouse melanoma cells (Figure 3A,B). We then treated fibroblast cells with Nb_4_C_3_T_*x*_ MXene and found that Nb_4_C_3_T_*x*_ MXene was also able to induce comets in a similar fashion (Figure 3C). After the start of the treatment, Ti_3_C_2_T_*x*_ steadily induced comets during 3 days of the experiment (Figure 4). Please note that the MXene was washed out after 4 h and cells, initially loaded with MXene, were actively proliferating, reaching a fully confluent monolayer (Figure 4B). Apparently, only a fraction of the MXene in cells initially loaded with MXene was present in the final monolayer population as a per cell amount. However, the MXene was able to induce comets. Calculations of the OTM of the comets showed no significant increase in this parameter only at time point 4 h (Figure 4C). However, careful visual inspection of the comets in the corresponding image (Figure 4A) endorsed a massive appearance of comets also at that time point but with shorter tails. This suggested that, at the early time points, the fragments of the chromosomal DNA were larger.

**Figure 3:**
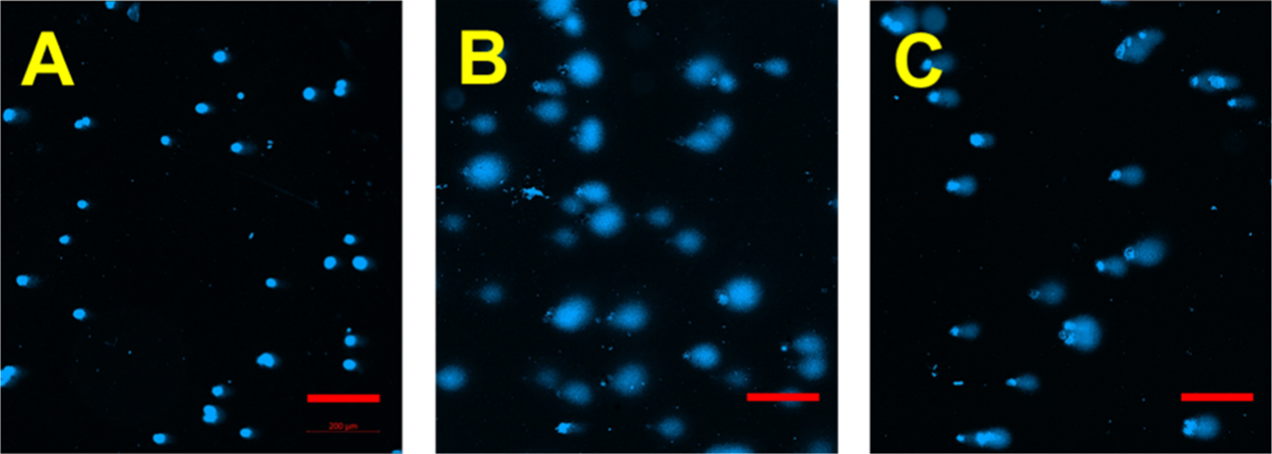
MXene-induced DNA comets in primary human fibroblast cells. The cells were treated with MXenes for 4 h, after which the MXene was washed away; the cells were further cultivated under normal conditions for 24 h and then subjected to the DNA comet assay. (A) control-untreated fibroblasts; (B) fibroblasts treated with Ti_3_C_2_T_*x*_ MXene; (C) cells treated with Nb_4_C_3_T_*x*_ MXene (concentration of both MXenes was 6.25 μg/mL, scale bars = 200 μm).

**Figure 4:**
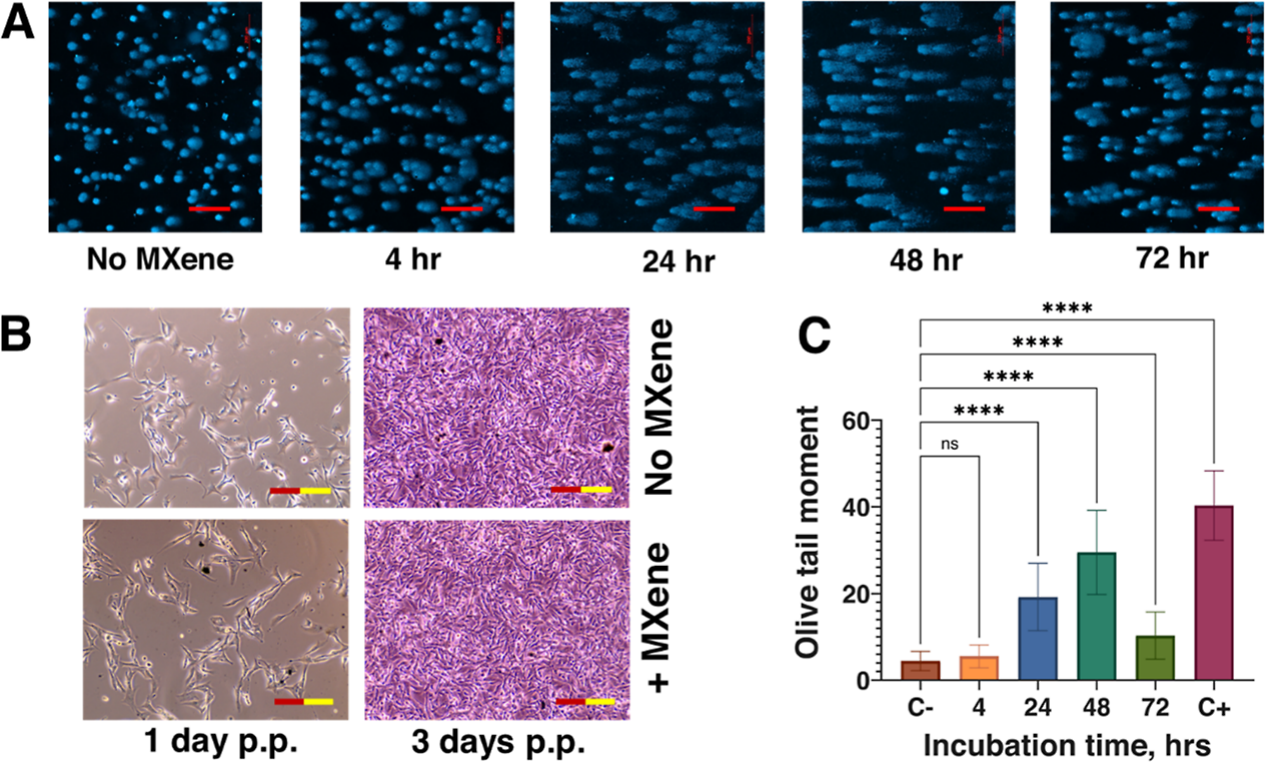
MXene-induced DNA comets from the point of treatment up to 3 days of cultivation. The mouse melanoma cells were loaded with 25 μg/mL of Ti_3_C_2_T_*x*_, after which the MXene was washed out, and the cells were further cultivated for 3 days postplating (p.p.). (A) DNA comet assays; (B) dark field microscopy images of the cells prior to trypsinization for the DNA comet assay; (C) results of the DNA comet assay presented as a graph where C- corresponds to no MXene control and C+ represents control with H_2_O_2_. Please note that a couple of dark inclusions at the “No MXene” control at 3 postplating (p.p.) in panel B look like MXene aggregates in the + MXene at 1 day p.p. but are, in fact, cell conglomerates because of the somewhat overgrown cell monolayer. The pinkish color of the images at 3 days p.p. is because the cells were already in the medium with resazurin. Scale bars = 200 μm.

We then investigated what quantity of MXene was enough to induce comets and found that MXenes induced comets at relatively low concentrations. Thus, under the described treatment scheme (4 h treatment with MXene followed by washing MXene out and further cultivation for 24 h), Nb_4_C_3_T_*x*_ MXene was able to induce comets at concentrations as low as 3.125 μg/mL (Supporting Information Figure S13). Even at lower concentrations, down to 1.56 μg/mL, it was possible to observe some cells with comets with both Ti_3_C_2_T_*x*_ and Nb_4_C_3_T_*x*_ MXenes in melanoma cells (not shown). We then addressed how long it takes for MXene to induce the comets. We treated the melanoma cells with 25 μg/mL of Nb_4_C_3_T_*x*_ and performed a time course experiment with the DNA comets. We found that Nb_4_C_3_T_*x*_ induced the comets 30 min after their addition to the melanoma cells (Supporting Information Figure S14). Altogether, this study suggested that MXenes-induced DNA comets under the alkaline DNA comet assay conditions were independent of the nature of MXenes and the type of cells.

### 3.4 MXenes Are Tolerated by the Cells and Do Not Induce Signs of Apoptosis and/or Necrosis

The robust induction of DNA comets by MXene also suggests the possibility of cell death. We counted slightly lower numbers of melanoma cells in wells, where the cells were treated with MXene (Supporting Information Table ST2). However, in line with our previous results and numerous observations by others,^50^ we did not detect any signs of cytotoxicity. Thus, the cells had a normal appearance, similar to the control untreated cells (the full panel, including cells treated for 4 h in serum-free medium, is shown in Supporting Information Figure S7). To quantify the viability of the MXene-loaded cells, we treated the melanoma cells with either Ti_3_C_2_T_*x*_ or Nb_4_C_3_T_*x*_ MXenes at various concentrations, including those that exceeded the concentrations used to induce DNA comets (6.25, 25, 50, and 100 μg/mL). We found that the MXene-loaded melanoma cells showed only a moderate reduction in their proliferative capacity (Figure 5A). A similar reduction in cell viability with both MXenes was observed also in primary fibroblast cells (Figure 5B), with a noticeably higher tolerance to Nb_4_C_3_T_*x*_ MXene.

**Figure 5:**
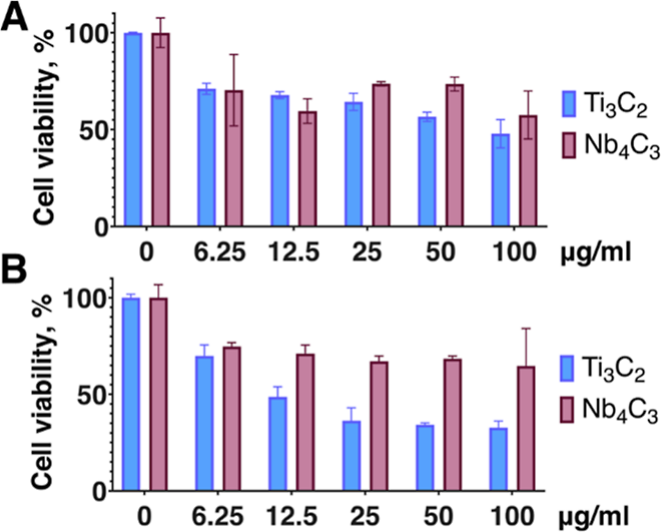
B16F10 mouse melanoma (A) and primary human fibroblast (B) cells were loaded with Ti_3_C_2_T_*x*_ and Nb_4_C_3_T_*x*_ MXenes at the indicated concentrations for 24 h and further incubated for 3 days, after which cell viability was accessed by the resazurin reduction assay. The data was normalized for the control values to compensate for differences in growth rates of different cell lines.

Moreover, the cells collected for the DNA comet assay after treatment did not show any apoptotic features (such as cell shrinkage, nuclear condensation, membrane blebbing, and formation of pyknotic bodies of condensed chromatin) or necrotic nuclei (karyolysis or karyorrhexis) when they were fixed, stained with DAPI, and observed under a fluorescent microscope (Figure 6).

**Figure 6:**
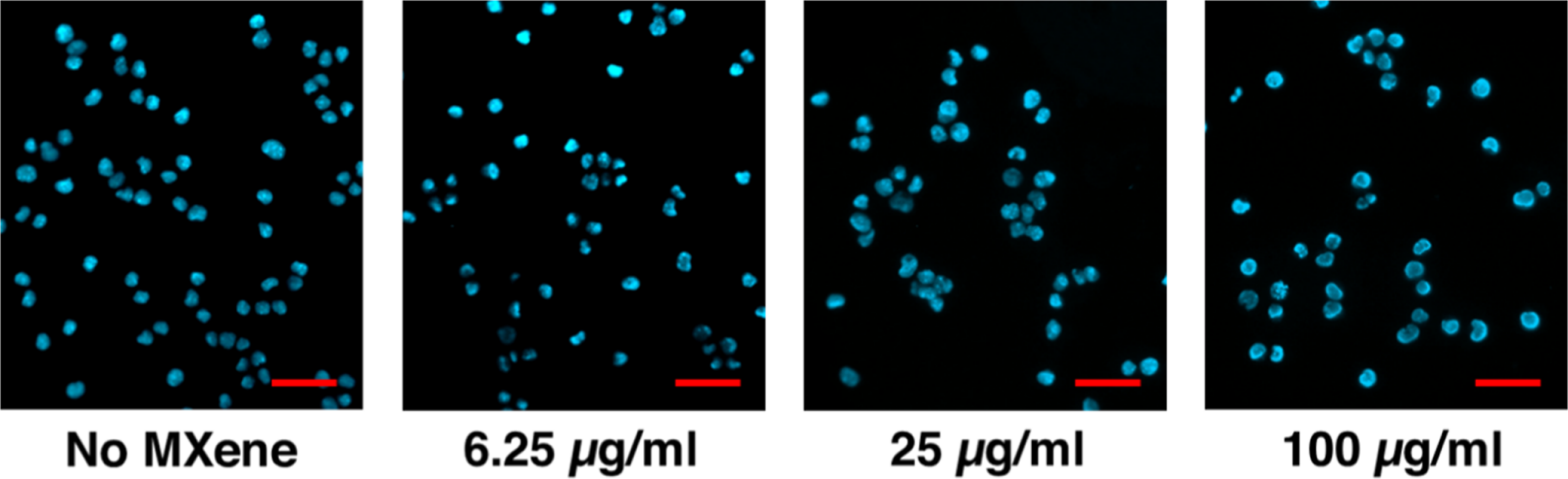
B16F10 mouse melanoma cells treated with MXene at the indicated concentrations for 4 h, followed by washing MXene out, changing medium, and further cultivation for 60 h under normal conditions. Aliquots of the cells collected for the DNA comet assay were fixed in methanol/acetic acid and stained with DAPI to visualize nuclei under a fluorescent microscope (magnification x400). Please note that these cells showed intensive DNA fragmentation manifested in the appearance of the DNA comets after they were subjected to the DNA comet assay. Scale bars = 50 μm.

To further investigate if MXene can induce apoptosis or necrosis in living cells, we stained the MXene-loaded melanoma cells with annexin V and PI and analyzed them by flow cytometry. We found that cells heavily loaded with MXenes (100 μg/mL of either Ti_3_C_2_T_*x*_ or Nb_4_C_3_T_*x*_) neither showed substantial signs of apoptosis nor necrosis even after prolonged incubation (Figure 7). We concluded that the fragmentation of chromosomal DNA and the development of DNA comets did not correlate with the normal appearance of the MXene-treated cells and the lack of signs of apoptosis and necrosis.

**Figure 7:**
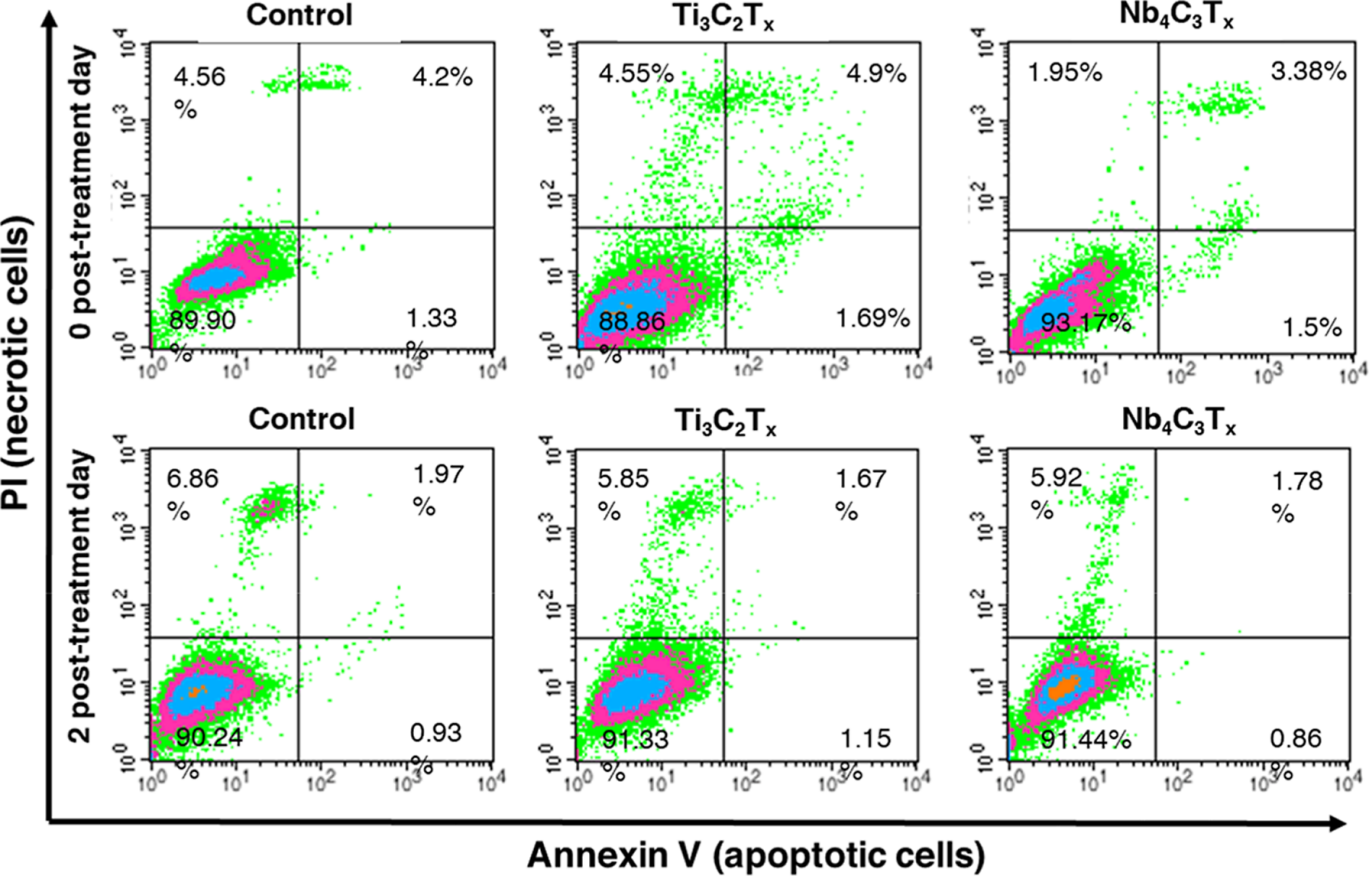
Flow cytometry assay with annexin V and PI labeled melanoma cells loaded with 100 μg/mL of Ti_3_C_2_T_*x*_ and Nb_4_C_3_T_*x*_ MXenes. Dot plots represent three independent flow cytometry measurements (*n* = 3).

### 3.5 Effect of Flake Size on the Ability of MXene to Induce DNA Comets

It was suggested that the MXene particle size might correlate with cellular uptake,^51^ although the mechanism behind this relationship has not yet been explored. To address the question of whether the size of the MXene flakes has any influence on the ability of MXene to induce DNA comets, fractionated Ti_3_C_2_T_*x*_ was added to the cells, and the cells were subjected to the alkaline DNA comet assay as before. We found that smaller fractions with an average size of 180 and 600 nm induced intensive DNA comets at a concentration of 25 μg/mL, similar to what we observed before (Figure 8A). However, MXene with a larger lateral size of 1000 and 3000 nm did not induce any visible DNA comets. To further verify if the manifestation of DNA comets correlates with cell viability, we loaded the melanoma cells with various fractions of Ti_3_C_2_T_*x*_ at different concentrations and accessed the cell viability using the resazurin reduction assay. We found that independently of the lateral size of the flakes, the MXenes led to a moderate decrement in cell viability, even at concentrations of MXene much higher than those that induced DNA comets (Figure 8B). A similar moderate effect of fractionated MXene was observed in primary fibroblast cells (not shown). We concluded that the impact of induction of the DNA comets by MXenes strongly depends on the size of the MXene flakes, where smaller flakes could induce comets, while the larger fractions could not induce any DNA comets. However, the effect of MXene on the cell viability does not depend on the flake size.

**Figure 8:**
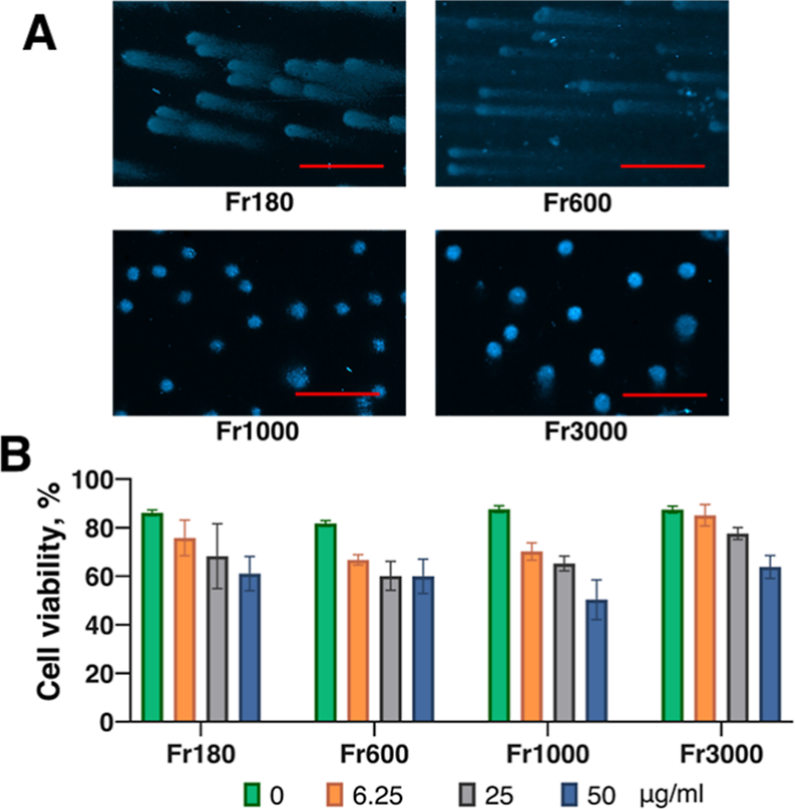
Effect of flake size on the formation of DNA comets. (A) fractionated Ti_3_C_2_T_*x*_ MXene with the flake size as indicated (fractions 180, 600, 1000, and 3000 nm) was applied to the DNA comet assay in B16F10 mouse melanoma cells at 25 μg/mL concentration under standard conditions; (B) B16F10 cells were loaded with fractionated Ti_3_C_2_T_*x*_ MXene at concentrations as indicated for 24 h, after which the cells were further incubated for 3 days, followed by the resazurin reduction assay. Scale bars = 200 μm.

A strong correlation between flake sizes and their ability to induce DNA comets suggested the hypothesis that smaller flakes could penetrate the cells while larger flakes could not. To investigate whether the flake size correlated with the ability of the smaller flakes to penetrate cells and localize in the nuclei, we loaded B16F10 mouse melanoma cells with 180 nm (small) and 3000 nm (large) fractions of Ti_3_C_2_T_*x*_ and studied their intracellular localization by TEM (Figure 9). We found that small MXenes could easily be observed in various cellular compartments, including nuclei (Figure 9C). In contrast, it was difficult to find flakes of the larger size both within and outside of the cells. With comparable effort, we found only one view field containing the 3000 nm MXene fraction (Figure 9F). The large flake was localized inside the cell, close to the nuclei, but not within the nuclei. We concluded that the large flakes were likely outside the cells at the moment of fixation and were washed away during fixation and contrasting procedures. Altogether, the TEM study confirmed that small-size MXene flakes were able to penetrate the cells and enter the nuclei, while larger flakes predominantly remained outside of the cells.

**Figure 9:**
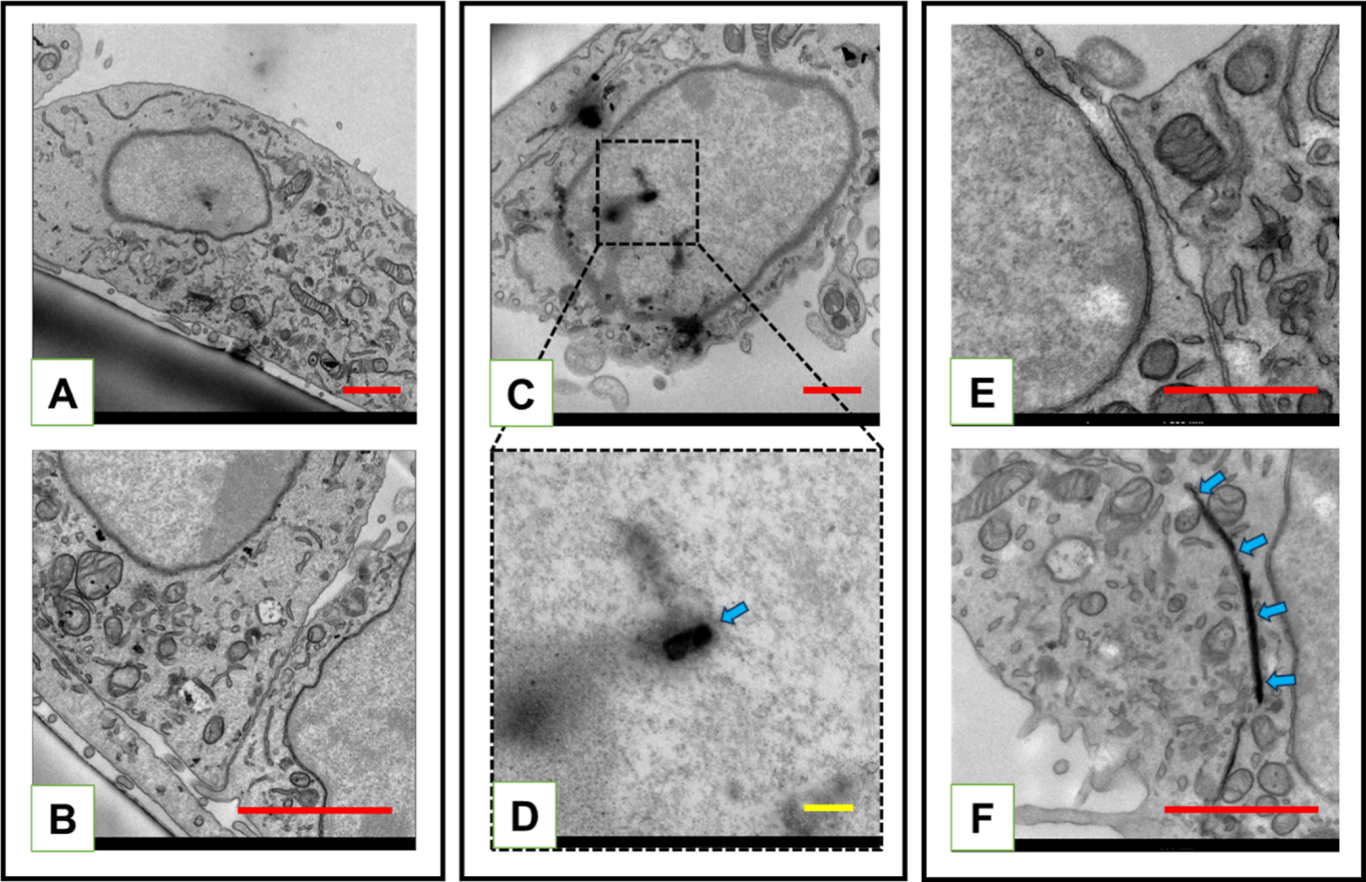
Analysis of B16F10 cells loaded with 180 and 3000 nm fractions of Ti_3_C_2_T_*x*_ MXenes by TEM. (A,B) Control cells (without MXenes) revealed a general distribution of organelles such as mitochondria, Golgi apparatus, and other membranous structures. The nucleus is well-defined, containing mostly heterochromatin with a continuous envelope. (B) Relationship between adjacent cells cultured under control conditions, indicating a well-established monolayer. (C) cells loaded with small (180 nm) MXene flakes demonstrated the accumulation of MXenes within the cytoplasm and nucleus. (D) Enlarged fragment of the nuclear space (framed with a dashed line), showing the interaction of a small MXene flake (arrow) with chromatin, which may contribute to DNA fragmentation. (E,F) Cells exposed to large (3000 nm) flakes showed a predominantly normal ultrastructural appearance without detectable MXenes. (F) Rarely seen large flake in the cytoplasm (arrows) in contact with mitochondria and membranous organelles, apparently without causing any major disturbances. Scale bars = 1 μm, except in D, where the scale bar is 200 nm.

### 3.6 MXenes Do Not Induce DNA Comets in Dead Cells

To check the possibility that the observed effect of the appearance of DNA comets was an artifact of the assay, we performed the DNA comet assay on metabolically inactivated cells. For this, we killed the cells in two different ways, namely, by incubation at 60 °C for 30 min and by incubation in 20% ethanol for 30 min, then incubated the cells with 25 μg/mL of Ti_3_C_2_T_*x*_ and proceeded with the DNA comet assay as usual. Metabolic inactivation of the cells was confirmed by a resazurin reduction assay (not shown). We observed that the incubation of the dead cells with MXene did not result in the appearance of DNA comets (Figure 10). We concluded that induction of DNA comets by MXenes requires alive metabolically active cells.

**Figure 10:**
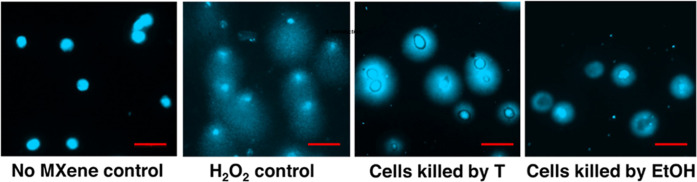
MXene does not induce DNA comets in dead cells. Cells were killed either by incubation at 60 °C for 30 min or by incubation with 20% ethanol for 30 min as indicated. After that, Ti_3_C_2_T_*x*_ MXene was added at 25 μg/mL, and the DNA comet assay was studied as previously described. Please note the absence of DNA comets in the dead cells. Scale bars = 50 μm.

### 3.7 Chromosomal DNA in MXene-Loaded Cells is Not Fragmented after Extraction

To investigate if the cells loaded with MXenes indeed have their chromosomal DNA fragmented due to strong interaction with MXenes, we treated the cells with the MXenes the same way as for the DNA comet assay and extracted the DNA by lyzing the cells and purifying the released DNA by phenol/chloroform extraction.^52^ We then analyzed the DNA with agarose gel electrophoresis. We found that the chromosomal DNA extracted from the cells treated with MXenes does not differ from the control DNA from the untreated cells (Figure 11). The fraction of the high molecular weight DNA remained unchanged, and the pattern of the lower molecular weight DNA fragments differed between treated and control samples. We concluded that the fragmentation of the chromosomal DNA in cells treated with MXenes and subjected to the DNA comet assay occurs by a mechanism independent of the mechanisms of maintaining DNA integrity in living cells.

**Figure 11:**
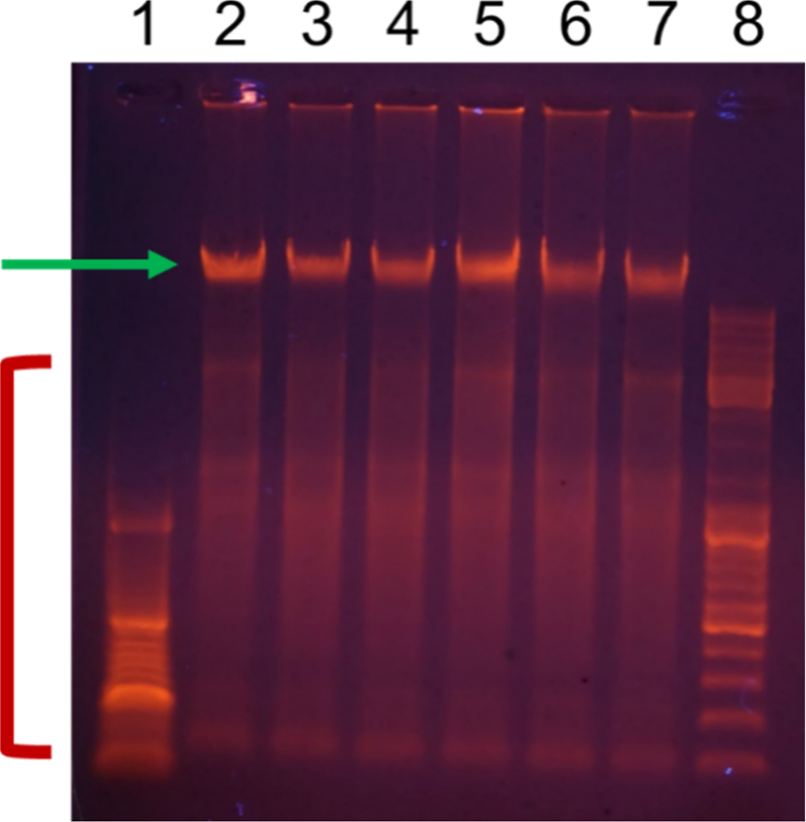
Agarose gel electrophoresis of the chromosomal DNA from B16F10 mouse melanoma cells (2 μg per lane) treated with Ti_3_C_2_T_*x*_ MXene similar to the treatment for the DNA comet assay. The position of the high molecular weight DNA is marked with a green arrow, while the position of the lower molecular weight DNA fragments is marked with a red bracket. Please note that the patterns of the lower molecular weight DNA fragments show no difference between the treated cells and the control untreated cells. 1–low molecular weight DNA marker (O’GeneRuler DNA Ladder 50–1000 bp, Thermo Fisher Scientific), 2- no MXene control, 3–cells treated with 6.25, 4–12.5, 5–25, 6–50, 7–100 μg/mL MXene, and 8–high molecular weight DNA marker (O’GeneRuler DNA Ladder Mix 100–10,000 bp, Thermo Fisher Scientific).

### 3.8 MXenes Do Not Cleave the Purified DNA under Conditions of the DNA Comet Assay

We hypothesized that the DNA could be fragmented via its physical interactions with the sharp edges of the MXenes under the electric field applied during the electrophoresis. MXene flakes have a higher modulus of elasticity and bending rigidity than rGO and other solution-processed 2D materials,^53,54^ so they can act as tiny knives capable of cutting through cell walls and biomolecules. To investigate this possibility, we reproduced the conditions of the DNA comet assay but with already purified chromosomal DNA instead of the MXene-loaded cells. For this, we mixed the DNA with various quantities of MXene and embedded it into low-melting-point agarose as if it were with the cells destined for the DNA comet assay. We then loaded the DNA/MXene/agarose mixtures into the wells of the agarose gel as if it were conventional agarose gel electrophoresis and performed the electrophoresis in the usual way (Figure 12). We found no increase in the amount of low molecular weight DNA fragments in samples where DNA was mixed with MXene. We concluded that the direct contact of high molecular weight DNA with MXene under the DNA comet assay conditions did not lead to DNA damage and fragmentation.

**Figure 12:**
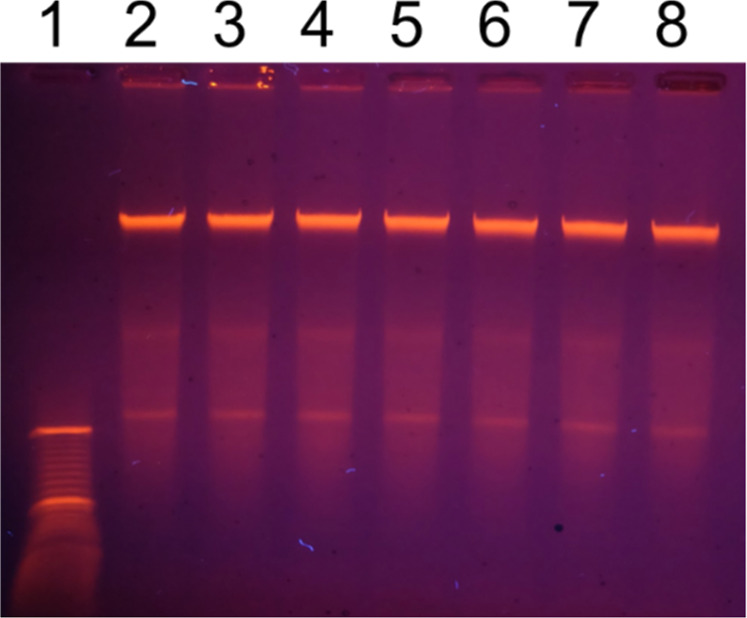
Purified chromosomal DNA does not undergo fragmentation by MXene under conditions of the DNA comet assay. One μg of the DNA was mixed with MXene in the ratio from 1:50 up to 1:1600, embedded into low-melting-point agarose (16 μL total volume of the sample), and loaded into agarose gel, followed by gel electrophoresis at 8 V/cm for 45 min. 1–low molecular weight DNA marker (O’GeneRuler DNA Ladder 50–1000 bp), 2–DNA with no MXene–control, 3–1 μg DNA mixed with 50 μg, 4–with 100 μg, 5–with 200 μg, 6–with 400 μg, 7–with 800 μg, and 8–with 1600 μg of Ti_3_C_2_T_*x*_.

### 3.9 Electrophoresis of DNA in Agarose Gel with MXene

We hypothesized that the DNA in the nuclei of the cells loaded with MXenes could be fragmented due to direct contact of the DNA with MXenes in the electric field under conditions of the DNA comet assay. To check this possibility, we made a gel of agarose with the same composition and concentration, which was normally used, but supplied with various quantities of MXenes (Supporting Information Figure S10). We then ran the chromosomal DNA of the mouse melanoma cells through an agarose gel with MXene under normal electrophoretic conditions (Figure 13). We found no increase in the amount of the low molecular weight DNA fragments after the high molecular weight chromosomal DNA passed through the agarose with MXene. The apparent change in the position of the band of the high molecular weight DNA in agarose with the maximum amount of MXene (lane 8) was most probably because of the distorted electric field due to the electrical conductivity of MXene. We concluded that high molecular weight chromosomal DNA did not undergo fragmentation while passing through the agarose gel with MXenes under the chosen experimental conditions.

**Figure 13:**
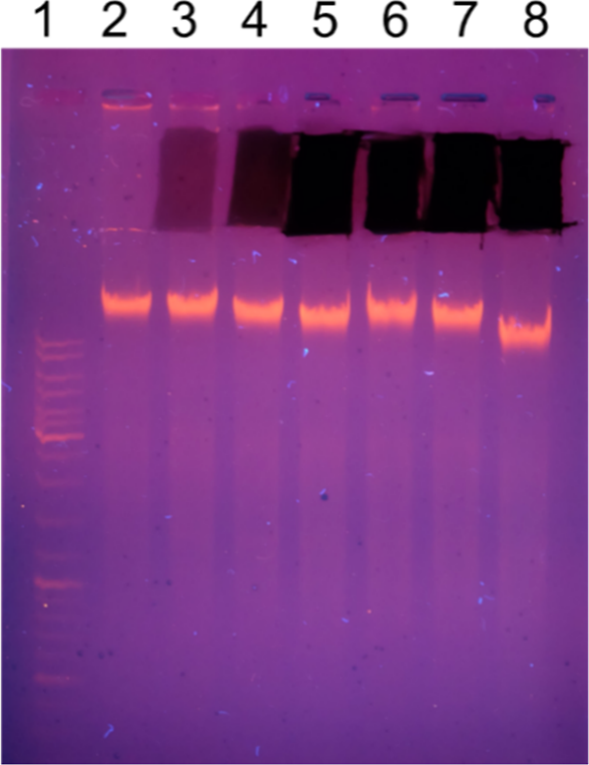
Intact chromosomal DNA from mouse melanoma cells (1.25 μg DNA per lane) runs through 0.8% agarose gel in 0.5× TBE buffer while passing through stretches of agarose of the same concentration and composition but supplied with various concentrations of Ti_3_C_2_T_*x*_ MXene. 1–high molecular weight DNA marker (O’GeneRuler DNA ladder mix 100–10,000 bp), 2–control lane without MXene, 3–0.07, 4–0.14, 5–0.28, 6–0.55, 7–1.1, and 8–2.2 mg/mL MXene. Electrophoresis was run for 1 h at 8 V/cm. Please note the slightly lower position of the DNA band in lane 8. We attributed the apparent increase in mobility of the DNA due to a distorted electric field on the agarose stretch with MXene due to the electrical conductivity of MXene in the gel.

### 3.10 In Vitro Electrophoresis

To verify if MXene can damage the chromosomal DNA within the cells in the electric field under conditions similar to those in the electrophoresis stage during the DNA comet assay but in living cells, we performed in vitro electrophoresis. We assumed that the DNA damage in living cells would result in changes in cell viability, and therefore, we performed a resazurin reduction assay immediately after in vitro electrophoresis. We also expected that if MXene indeed can cause damage to DNA in the electric field under conditions of the electrophoresis, lysing of the cells, and extraction of the DNA immediately after the in vitro electrophoresis will result in visible degradation of the high molecular weight DNA.

We found that cells indeed showed lower viability after in vitro electrophoresis for 10 min (Figure 14A), most probably because of changes in pH due to the applied potential and passing electric current. However, the presence of Ti_3_C_2_T_*x*_ MXene did not result in a lowered viability of the cells. Nb_4_C_3_T_*x*_ MXene led to diminished viability of the cells after in vitro electrophoresis (not shown). Likewise, the smallest fraction of Ti_3_C_2_T_*x*_ MXene (Fr180 for 180 nm flake size), which was active in inducing DNA comets, did not lead to diminished cell viability after in vitro electrophoresis in the presence of 25 μg/mL Fr180 (not shown). Moreover, chromosomal DNA extracted immediately after in vitro electrophoresis for 10 and 20 min showed no increased fragmentation due to the presence of MXene (Figure 14B).

**Figure 14:**
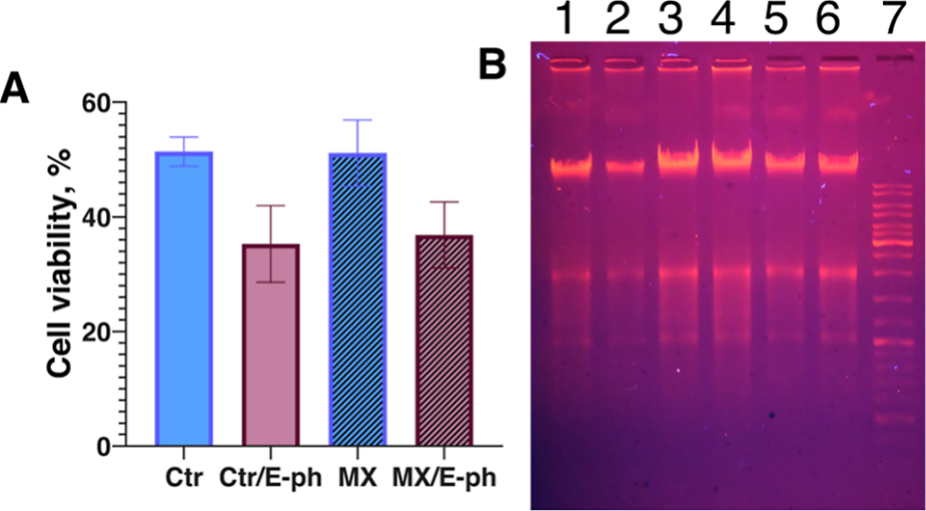
In vitro electrophoresis of melanoma cells loaded with 6.25 μg/mL of Ti_3_C_2_T_*x*_ MXene. (A) Resazurin reduction assay shows that the presence of MXene does not lead to the diminished viability of the cells under conditions of in vitro electrophoresis; (B) 1 μg of chromosomal DNA from mouse melanoma extracted immediately after electrophoresis was analyzed in 0.8% agarose gel in 0.5× TBE buffer. Ctr and MX–control cells and cells with MXene, respectively, without electrophoresis (samples with MXenes are marked with the filled columns); Ctr/E-ph and MX/E-ph-control cells and cells with MXene, respectively, after electrophoresis; 1,2-DNA from control cells and cells with MXene, respectively, after 10 min electrophoresis; 3,4-DNA from control cells and cells with MXene, respectively, without electrophoresis; 5,6-DNA from control cells and cells with MXene, respectively, after 20 min electrophoresis; 7-molecular weight DNA marker (GeneRuler DNA Ladder Mix, 100 to 10,000 bp).

We assumed that the charged MXene flakes could move in the strong electric field, leading to DNA fragmentation and hence diminishing cell viability. The melanoma cells were loaded with 6.25, 50, and 100 μg/mL of Ti_3_C_2_T_*x*_ MXene for 4 h, and the whole plate was placed between the electrodes 30 cm apart at 20,000 V potential. We found that applying such a potential for 20 min did not change cell viability (not shown).

## 4 Discussion

Careful examination of the available data suggests that accurate toxicity profiles for both Ti and Nb MXenes are still elusive, with contradictory findings even for the same chemical formulations of MXenes. This inconsistency can be attributed to various factors such as chemical purity, oxidation state, and terminations of the MXenes employed. As of today, only a limited portion of biomedical research adequately considers all possible variables, including surface chemistry, flake structure, oxidation state, and *T*_*x*_ terminations. For instance, fluorine terminations can lead to hydrolysis in aqueous solutions, yielding hydrofluoric acid (HF),^55^ which can substantially impact biocompatibility. Improper storage of MXenes can also induce their oxidation,^56^ resulting in the formation of titanium dioxide, which, in turn, can affect cell viability. Chemical purity, specifically incomplete removal of AlF_3_, HF, or LiCl during the delamination process,^57^ can directly influence cell responses, potentially generating flawed results. MXene flake size is another factor influencing cell viability, though research in this area is still limited. The incubation duration with cells further complicates the interpretation of results, as existing data show a wide range of coincubation times, from 6 h to 7 days, making direct comparisons difficult. Shorter incubation periods have less impact on cells when compared to extended coculturing.

Moreover, the choice of biochemical assay methods, such as MTT, CCK-8, and resazurin, for evaluating cell viability can also affect results and their interpretation. For instance, we have previously demonstrated that Ti_3_C_2_T_*x*_ can cause the autoreduction of resazurin, potentially leading to false-positive findings.^11^ To navigate this complexity and gain a precise understanding of MXene biocompatibility at the cellular level, rigorous control and standardization of these factors are imperative. Considering all of these factors, it is paramount to thoroughly investigate the potential genotoxicity of MXenes prior to their widespread use in biomedical applications.

Genotoxic effects can be incurred either by direct interaction of the agent with the DNA or by interfering with DNA maintenance and repair mechanisms.^58^ In the case of MXenes, with their yet unknown genotoxic effects, their interaction with genetic materials has remained an open question. We found that Ti_3_C_2_T_*x*_ MXene induced apparent DNA fragmentation in living mouse melanoma cells in a concentration- (Figure 2) and size-dependent manner (Figure 8) in DNA comet assays. Ti_3_C_2_T_*x*_ induced the appearance of the DNA comets in cells in contact with various concentrations of MXene for 4 h and further grown for up to 3 days. Although the MXene was washed away from the cell surface, the cells underwent multiple expansions from a sparse coverage to a confluent monolayer (Supporting Information Figures S4, S5, and S7), and the cells still produced robust DNA comets (Figure 2). It suggested that small-size MXene was able to induce DNA comets in melanoma cells even at marginal concentrations. We then addressed the ability of MXenes to induce apparent DNA fragmentation in both immortalized cancer cells, which have a genetic background allowing them to proliferate indefinitely, and primary cells with a limited proliferation potential. Accordingly, we investigated if the observed effect was cell-specific and found that Ti_3_C_2_T_*x*_ could also induce DNA comets in human fibroblast cells (Figure 3B). We then checked if the observed effect was MXene-specific and found that the Nb_4_C_3_T_*x*_ MXene was also able to induce DNA comets (Figure 3C). The MXene-loaded cells continuously developed DNA comets for up to 3 days of the experiment. The initially sparse MXene-loaded cells reached a confluent monolayer, but almost all cells in the population developed comets (Figure 4). Development of the DNA comets required relatively low concentrations of MXene −3.25 μg/mL of the Nb_4_C_3_T_*x*_ was enough to effectively induce DNA comets (Supporting Information Figure S13). It was possible to observe some comets even at half of that concentration, at which the presence of MXenes was barely visible. Induction of DNA comets occurred relatively quickly. Thus, the cells loaded with 25 μg/mL of Nb_4_C_3_T_*x*_ developed the comets already after 30 min of incubation (Supporting Information Figure S14). Altogether, this suggests that MXenes can induce DNA fragmentation in cell types with very different genetic backgrounds when placed in an electric field. Primary human cells were used to investigate the potential genotoxicity of MXenes due to the fact that forthcoming biomedical applications of MXenes presume direct contact with cells and tissues in the human body and thus with the “primary” cells.

Strikingly, MXene did not induce any substantial cytotoxicity (Figure 6). Moreover, even cells heavily loaded with MXenes did not induce apoptosis or necrosis (Figure 7). Neither Ti_3_C_2_T_*x*_ nor Nb_4_C_3_T_*x*_ induced any profound cytotoxic effect even after prolonged incubation post-treatment at concentrations 2 orders of magnitude higher than the concentrations at which the DNA comets robustly appear. It is already established that MXenes are generally well tolerated by the living systems.^59^ On the other hand, the appearance of the DNA comets suggests fragmentation of nuclear DNA manifested and a potent genotoxic effect, which should be accompanied by extensive cytotoxicity. This discrepancy raised an assumption that the observed DNA comets were an artifact of the DNA comet assay. We addressed that possibility by treating metabolically inactivated cells with MXene. Although the dead cells appeared different under the DNA comet assay conditions than normal live cells, their DNA was still visible, but we did not observe any DNA comets (Figure 10).

DNA could potentially be shredded by contacting the edges of the nanometer-thin MXene flakes. Graphene oxide and other 2D materials have sharp edges.^60^ Mechanical damage to the cell walls of bacteria^61^ and membranes of eukaryotic cells^62^ has been described as a factor behind the antibacterial properties of MXenes and hemolysis of red blood cells. MXene was reported to be much less damaging to the red blood cells and more biocompatible than graphene oxide,^62^ despite its higher rigidity than GO, rGO, and other common 2D materials. Nevertheless, MXenes theoretically can cut DNA strands by the edges of their flakes. This scenario could be especially appropriate under DNA comet assay conditions, where the DNA and MXene are placed in the electric field, prompting them to move. First, we investigated whether the chromosomal DNA was fragmented in the cells treated similarly to the DNA comet assay. We extracted the genomic DNA from the mouse melanoma cells, which were treated with MXene and were destined for the DNA comet assay. We found that the DNA in the MXene-loaded cells was intact (Figure 11). We then addressed the question of the movements of the DNA mixed with MXene and placed the mixture into an electric field under conditions of electrophoresis that could damage the DNA strands. The purified chromosomal DNA was mixed with various quantities of MXene, embedded in the mixture into low melting point agarose, as if those were MXene-loaded cells intended for the DNA comet assay, loaded in the electrophoresis gel, and ran electrophoresis. We did not observe any DNA fragmentation by MXene under the gel electrophoresis conditions (Figure 12). We then opted to verify the possibility that the DNA could be fragmented while moving past the MXene flakes. For this, we made an agarose gel, in which parts of the gel contained various quantities of MXene. We ran the intact chromosomal DNA through the MXene-containing gel and found that the DNA did not get fragmented while passing through the MXene-containing environment (Figure 13).

To further verify whether the sharp MXene flakes could trigger DNA fragmentations under gel electrophoresis in living cells, we developed an in vitro electrophoresis technique. We loaded the cells with MXene and ran electrophoresis directly in the cell culture wells. We found that the presence of MXene did not result in an additional drop in cell viability under the electrophoresis conditions (Figure 14A). Moreover, the DNA extracted from the MXene-loaded cells immediately after in vitro electrophoresis did not show signs of degradation (Figure 14B). The strong electric field (66.7 kV/m) neither resulted in diminished cell viability. We concluded that the applied electric field was not the reason for the fragmented DNA seen in the DNA comet assay.

The differences in the properties of MXenes with the same nominal chemical formula can explain the discrepancies and somewhat controversial biomedical properties of the MXenes published so far. We showed it using the flake size effect as an example in this study. Moreover, we hypothesized that the “sharpness” of MXenes depends on their age, as oxidation leads to oxide nanocrystals forming along the edges.^48^ However, we did not find any differences in the properties of the “old” and the “fresh” Ti_3_C_2_T_*x*_ MXene stocks in several different experiments (not shown). While the chemistry, structure, and properties of MXenes depend on their manufacturing, delamination, and storage conditions,^5,50^ the fact that M_3_C_2_T_*x*_ and M_4_C_3_T_*x*_ MXenes, fresh or oxidized, with Ti or Nb metal on the surface all caused similar impact, suggests that mechanical rather than chemical effects caused the observed DNA comets.

Considering that all of our experiments conducted to test the initial hypothesis demonstrated no cytotoxicity or DNA damage, the only explanation left is that the DNA and cell walls were cut by MXene flakes in the DNA comet assays and only when the flakes were inside the cells, with cells immobilized in gel and unable to move along with MXene. Figure 8 shows that micrometer-sized or larger flakes that cannot cross cellular membranes do not cause comets. Figure 10 shows that dead cells in which the endocytosis mechanisms do not work were unaffected by MXenes and did not produce DNA comets. Also, freely suspended MXene can move around the cells or DNA or along with them, not causing any damage, and DNA can move through the gel with MXenes without being shredded in pieces (Figures 11–14). Negatively charged MXene flakes orient along the electric field and move toward the positively charged electrode.^63^ When they can move in solution, they float along DNA or cells with the surrounding environment (cell, protein corona, etc.). However, when a cell is immobilized in gel and MXene flakes are trapped inside, they will still rotate and move, shredding DNA and other large biomolecules inside the cell and cutting through the cell walls. This is the only mechanism that does not contradict any of the experiments conducted. Of course, in situ observation of MXenes flakes movement is highly desired to provide direct evidence, but it is particularly difficult to achieve by optical means for nanometer-thin flakes with a lateral size of tens or a couple of hundred nanometers.

The mechanism of the observed MXene-induced DNA comet phenomenon still requires further investigation. Further research is also needed to understand to what extent the observed DNA fragmentation of cells embedded in the gel has any biologically meaningful consequences, especially concerning processes related to genome stability, cell division, and integrity of genes such as oncogenes or tumor-suppressor genes. Although the cultured cells were not affected in our in vitro experiments when exposed to the electric field, cells in living tissues may respond differently under such conditions. In tissues, cells are confined within the extracellular matrix, making them somewhat similar to cells embedded in the agarose gel during a DNA comet assay. Therefore, electrophoresis or exposure to a strong electric field from equipment or power lines may present a risk for a person with MXene flakes introduced into the tissue for drug delivery, imaging, or photothermal therapy. This will require in vivo studies. Ti_3_C_2_T_*x*_ and Nb_4_C_3_T_*x*_ have been widely studied as photothermal therapy agents due to their absorption bands in the near-IR and IR range, respectively. At the same time, an electric field may be used instead of infrared light to destroy tumors loaded with MXenes. This may be advantageous for treating tumors located much deeper than the light can penetrate. Overall, the in vitro findings reported in this study of two popular MXenes will support the translation of MXene research into healthcare practices.

## 5 Conclusions

DNA comet assay experiments showed robust fragmentation of chromosomal DNA in cells loaded with MXenes and embedded in the gel. Ti_3_C_2_T_*x*_ and Nb_4_C_3_T_*x*_ MXenes of less than 1 μm in lateral dimensions induced DNA comets in mouse melanoma and human fibroblast cells. Larger flakes did not induce DNA fragmentation or comet formation. The comet formation did not depend on the MXene structure or chemistry, and the fact that only the flake size played a role suggests that endocytosis is required to form comets. Unless MXene particles are inside the cells, no DNA comets are formed.

Despite the findings of DNA comets, cells loaded with MXenes and destined for the DNA comet assay showed no signs of cytotoxicity. Moreover, the extraction of chromosomal DNA from the MXene-loaded cells showed no visible DNA fragmentation. Modulating the DNA comet assay conditions with purified chromosomal DNA mixed with MXenes instead of the MXene-loaded cells did not show any DNA damage either. Finally, electrophoresis conducted on living cells loaded with MXene did not demonstrate direct DNA cleavage by the sharp edges of the MXene flakes. These experiments showed excellent biocompatibility of titanium and niobium carbide-based MXenes.

We demonstrated that the most probable mechanism of DNA comet formation is the rotation and movement of submicrometer MXene flakes inside cells in the electric field. This leads to cleavage and shredding of DNA by MXene’s razor-sharp edges. This suggests a potential risk of the smallest MXene particles being able to penetrate through the cell walls. However, the risk exists only if the person is subjected to a strong electric field capable of moving those particles through electrophoresis. On the other side, this finding opens the opportunity for replacing or combining photothermal therapy with electrophoretic cancer treatment using the already developed mechanisms for MXene particle delivery.

## Supplementary Material



## Notes

DLS of fractionated Ti_3_C_2_T_*x*_ MXene with various flake sizes; zeta potential of Ti_3_C_2_T_*x*_ MXene fractionated by flake size; dynamic UV–vis spectra of Ti_3_C_2_T_*x*_ MXene with various flake sizes; XRD patterns of Nb_4_C_3_T_*x*_ MXene and its precursor Nb_4_AlC_3_; B16F10 mouse melanoma cells incubated with Ti_3_C_2_T_*x*_ MXene; B16F10 cells incubated with Ti_3_C_2_T_*x*_ MXene after washing MXene out and changing the medium; working dilutions of Ti_3_C_2_T_*x*_ MXene in medium with serum and in serum-free medium; cell counts of Ti_3_C_2_T_*x*_ MXene treated melanoma cells prior to the DNA comet assay; Ti_3_C_2_T_*x*_ MXene treated cells after prolonged cultivation for 60 h; examples of the image processing by the Open Comet software; DNA extraction from the Ti_3_C_2_T_*x*_ MXene-loaded cells; agarose gel with Ti_3_C_2_T_*x*_ MXene; 3D printed inserts for in vitro electrophoresis; SEM and TEM images of MXenes; ability of Nb_4_C_3_T_*x*_ MXene to induce comets at low concentrations down to 1.5 μg/mL; and ability of Nb_4_C_3_T_*x*_ MXene to induce comets already after 30 min post treatment (PDF)
