# Supplementary material for: Elucidation of Potential Genotoxicity of MXenes Using a DNA Comet Assay
Source: ACS Appl Bio Mater. 2024 Dec 3;7(12):8351–66. doi: 10.1021/acsabm.4c01142 (PMC12239077; doi:10.1021/acsabm.4c01142)
Supplement: Supplementary file 1 [file mt4c01142_si_001.pdf]

Supporting Information

Elucidation of Potential Genotoxicity of MXenes Using a DNA Comet Assay

Sergiy Kyrylenko[1], Inna Chorna[1], Zhanna Klishchova[1,2], IlyaYanko[1], Anton Roshchupkin[1], Volodymyr Deineka[1,3], Kateryna Diedkova[1,3], Anastasia Konieva[1,4], Oksana Petrichenko[3], Irina Kube-Golovin[4], Gunther Wennemuth[4], Emerson Coy[7], Iryna Roslyk[5,6], Ivan Baginskiy[5], Veronika Zahorodna[5], Oleksiy Gogotsi[5], Benjamin Chacon[6], Luciana P. Cartarozzi[8], Alexandre L. R. Oliveira[8], Igor Iatsunskyi[7], Yury Gogotsi[6], Maksym Pogorielov[1,3*]

[1] Biomedical Research Center, Sumy State University, 31 Sanatorna St, Sumy 40007, Ukraine

[2] Federal University of Lavras UFLA, Lavras, Minas Gerais, CEP 37203-202, Brazil

[3] University of Latvia, Institute of Atomic Physics and Spectroscopy, 3 Jelgavas St, Riga LV-1004, Latvia

[4] Department of Anatomy, University Hospital, University Duisburg-Essen, Hufelandstr. 55, D-45147 Essen, Germany

[5] Materials Research Centre, 3 Krzhizhanovskogo St, Kyiv 03680, Ukraine

[6] A.J. Drexel Nanomaterials Institute and Departmental of Materials Science and Engineering, Drexel University, 3141 Chestnut St, Philadelphia, PA 19104, United States

[7] NanoBioMedical Centre, Adam Mickiewicz University, 3, Wszechnicy Piastowskiej Str., 61-614 Poznan, Poland

[8] Laboratory of Nerve Regeneration, Institute of Biology, University of Campinas, Campinas, SP, 13083-862, Brazil

[*]Address correspondence to: m.pogorielov@gmail.com

Supporting Information

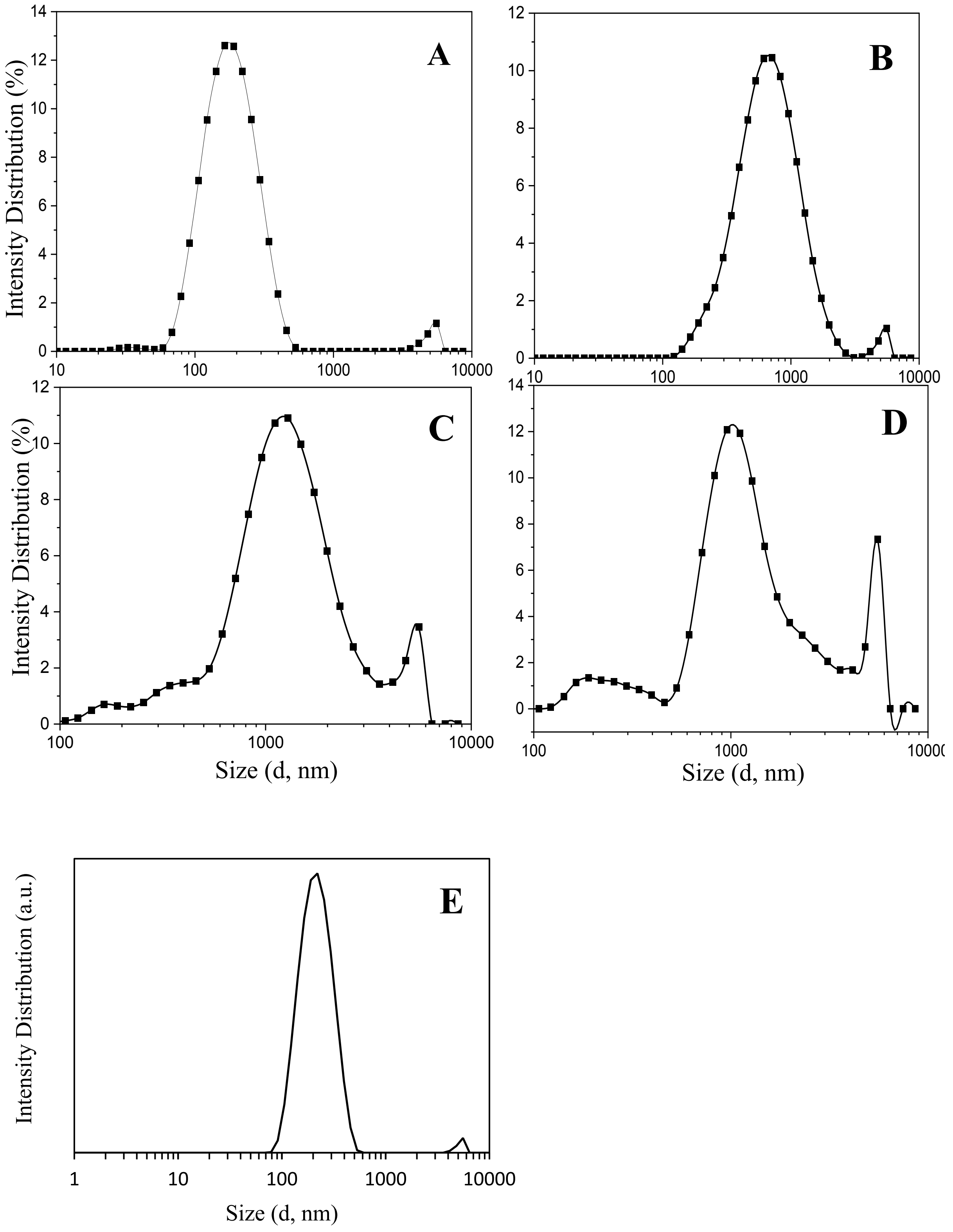

Supplementary Figure S1. Dynamic light scattering (DLS) intensity distribution of fractionated $Ti_3C_2T_x$ MXene with average flake size (A) 150 nm, (B) 600 nm, (C) 1000 nm, and (D) 2000 nm. (E) non-fractionated $Nb_4C_3T_x$ MXene.

Supporting Information

Supplementary Table ST1. Zeta potential of $Ti_3C_2T_x$ MXene fractionated by flake size.

Fraction #	Average size, nm	Zeta potential, mV
1	150	-50.0
2	600	-44.5
3	1000	-48.8
4	2000	-49.5

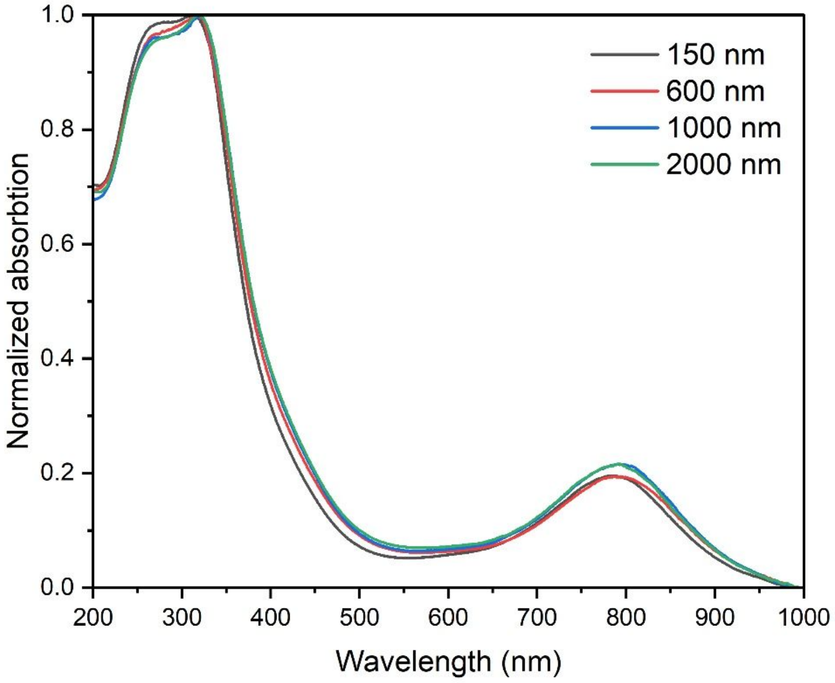

Supplementary Figure S2. Dynamic UV-vis spectra of $Ti_3C_2T_x$ MXene samples with various flake sizes.

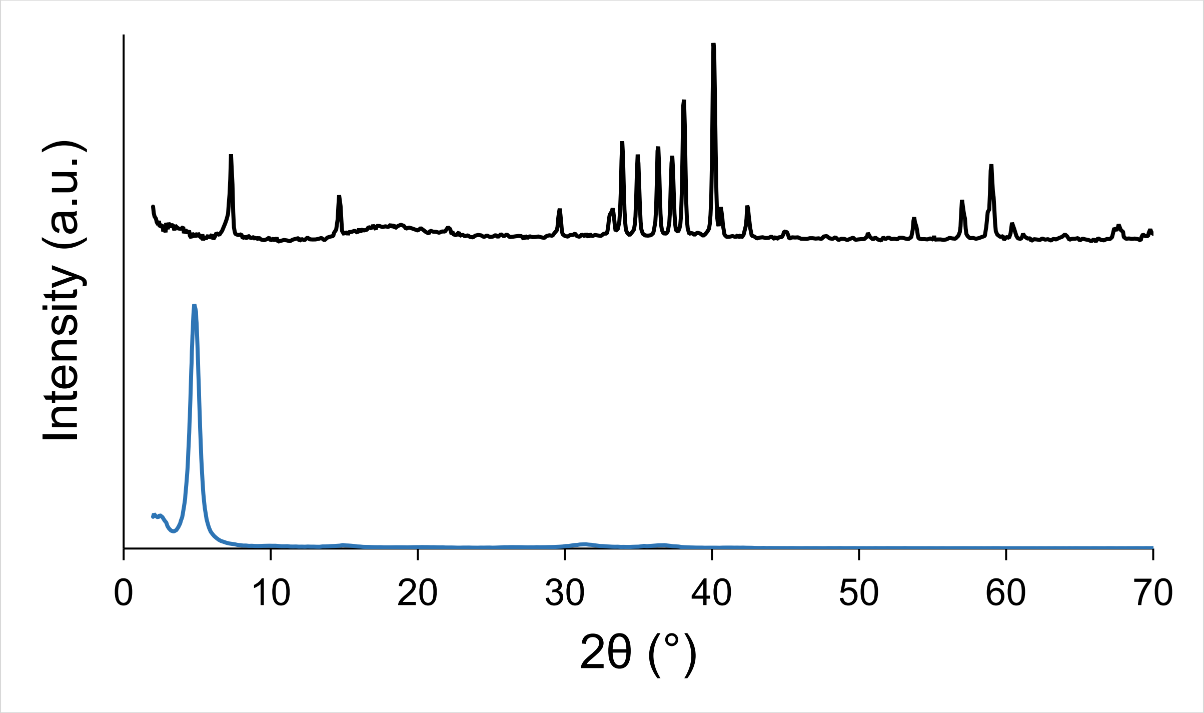

Supplementary Figure S3. XRD patterns of $Nb_4C_3T_x$ MXene (blue) and its precursor Nb_4AlC_3 (black)

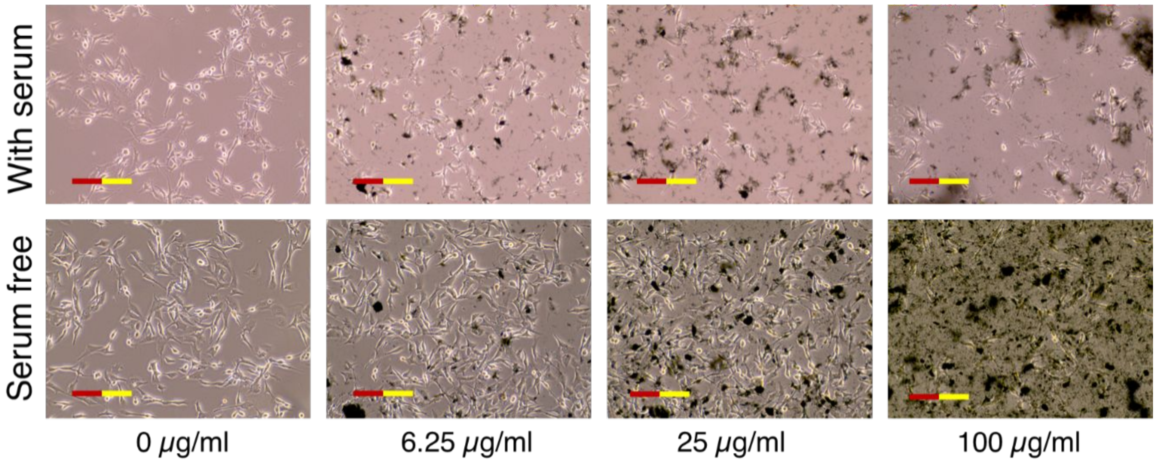

Supplementary Figure S4. B16F10 mouse melanoma cells incubated for 4 hrs with $Ti_3C_2T_x$ MXene (before washing MXene out and changing medium). Scale bar = 200 μm.

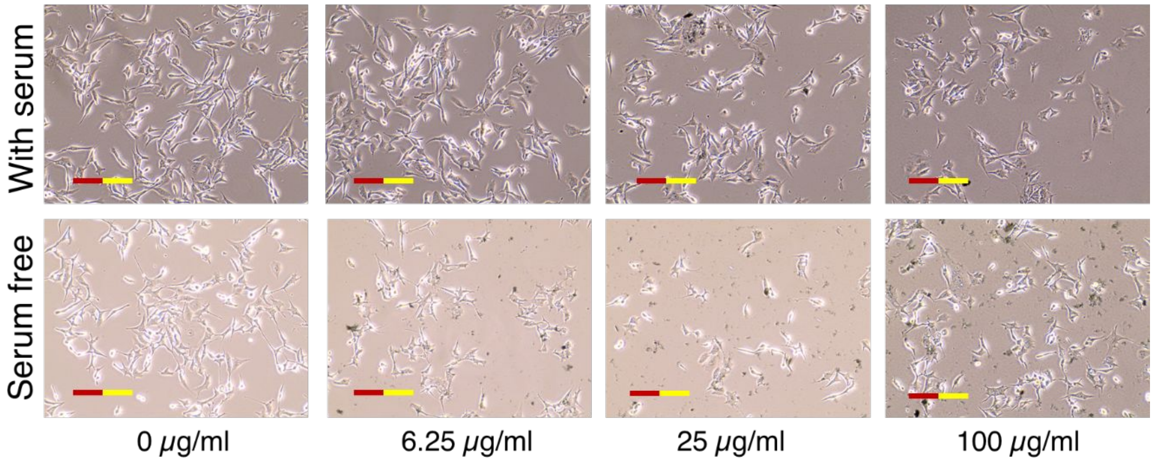

Supplementary Figure S5. B16F10 mouse melanoma cells incubated for 4 hrs with $Ti_3C_2T_x$ MXenes after washing MXene out and changing the medium. Scale bar = 200 µm.

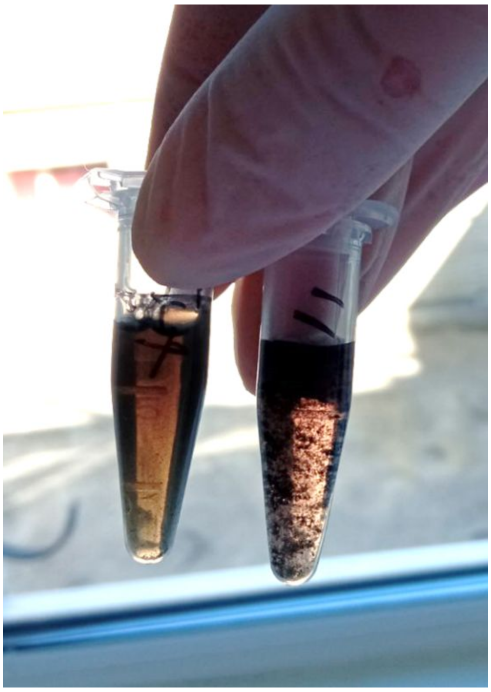

Supplementary Figure S6. Working dilutions of $Ti_3C_2T_x$ MXene before adding to the cell plates, to the left – in the medium with serum, to the right – serum-free.

Supporting Information

Supplementary Table ST2. B16F10 mouse melanoma cells were treated with $Ti_3C_2T_x$ MXene for 4 hrs in complete medium and incubated for additional 2 days as described, after which the cells were taken by trypsinization and counted, just prior to the DNA comet assay.

	Cells per well
Untreated control	3.1 mln
6.25 µg/ml	0.69 mln
25 µg/ml	0.61 mln
100 µg/ml	0.4 mln

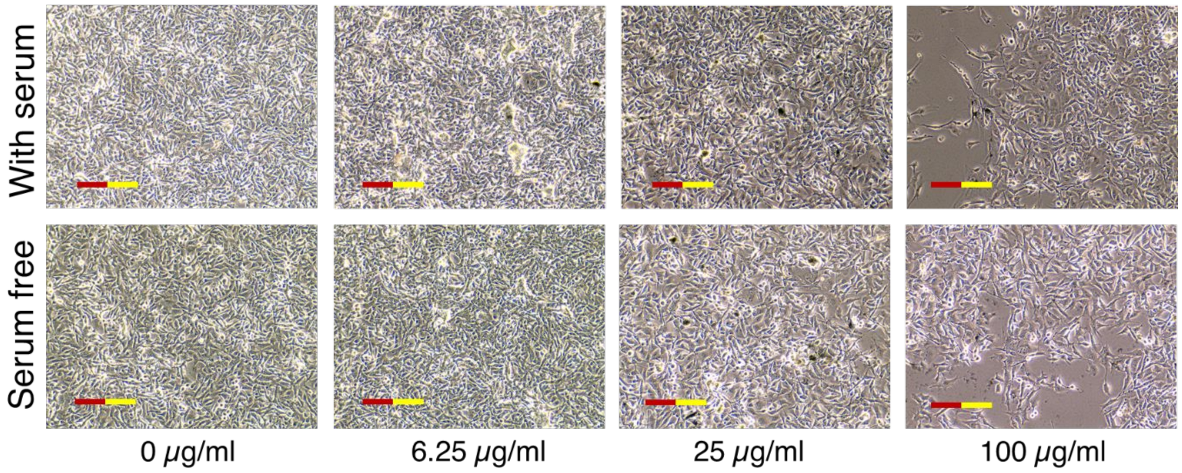

Supplementary Figure S7. B16F10 mouse melanoma cells, treated with $Ti_3C_2T_x$ MXene for 4 hrs followed by washing MXene out, changing medium and cultivating further for 60 hrs. Images were taken just before trypsinization of the cells for the DNA comet assay. Scale bar = 200 µm.

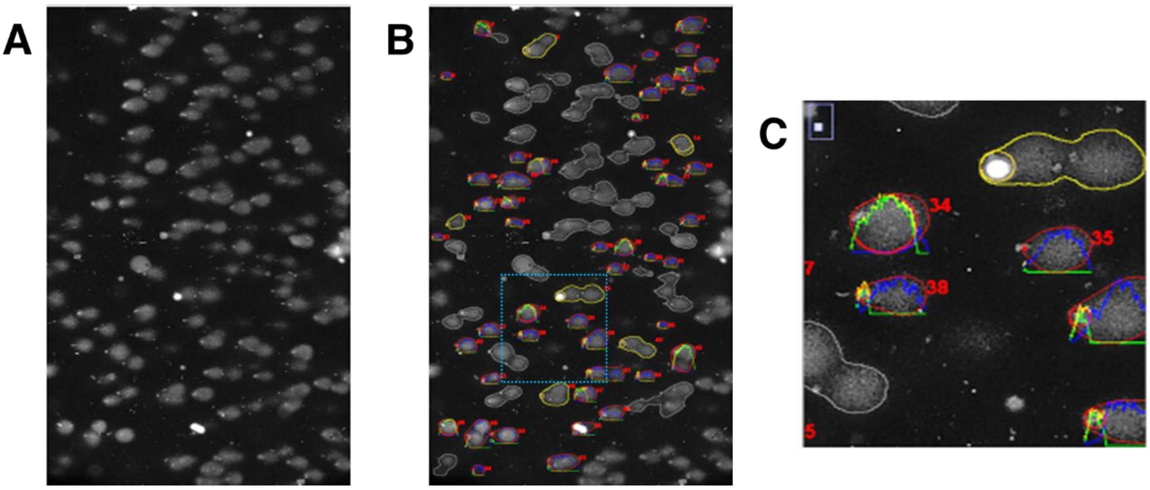

Supplementary Figure S8. Example of the image processing by the Open Comet software. A, initial image of the DNA comet assay; B, the same image with program-made identifications applied; C, enlarged section of the image as shown with a blue dotted rectangle in B. The enlarged version displays several comet identifications generated by the program. In this example, the comet with the assigned #38 was taken into account, while the comet #34 and #35 were considered defective and were excluded from calculations. The comet with a bright white spot (apparently a dust particle) in the upper right corner, outlined by a yellow line, was neither taken into account.

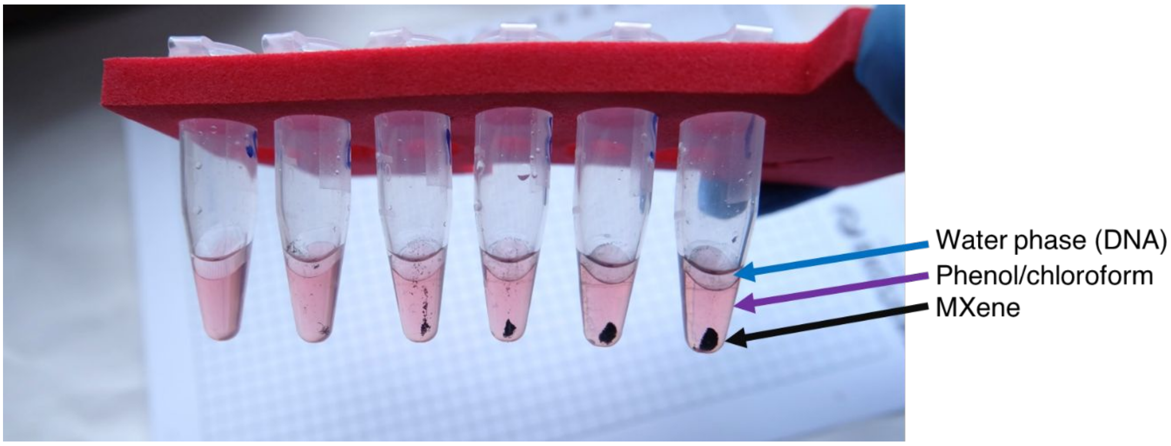

Supplementary Figure S9. DNA extracted from the cells loaded with $Ti_3C_2T_x$ MXene is essentially free from the traces of MXenes. The image confirms that the procedure of phenol/chloroform extraction effectively removes MXenes. Please note precipitated MXenes on the bottoms of the tubes after centrifugation.

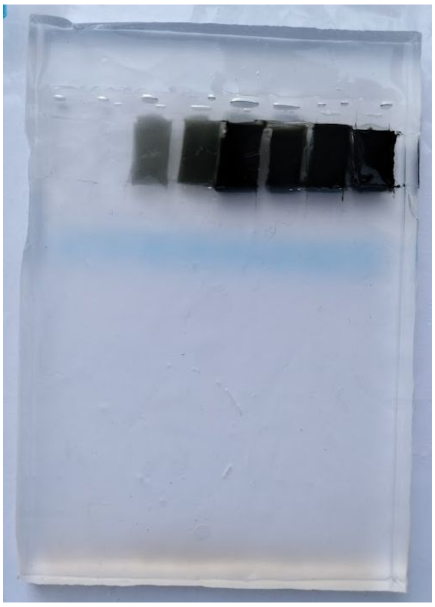

Supplementary Figure S10. The fragments of the gel 6x10 mm were cut out of the normal 0.8% agarose gel in 0.5x TBE, and the space was filled with the agarose of the same concentration and composition but supplied with various quantities of $Ti_3C_2T_x$ MXene (from 69 µg/ml up to 2.2 mg/ml with 2x increment). The intact chromosomal DNA from mouse melanoma cells was loaded into the wells and the electrophoresis was run as usual at 8 V/cm for 55 min, followed by EtBr staining and UV visualization. The image of the gel was taken in visible light after gel electrophoresis and UV visualization.

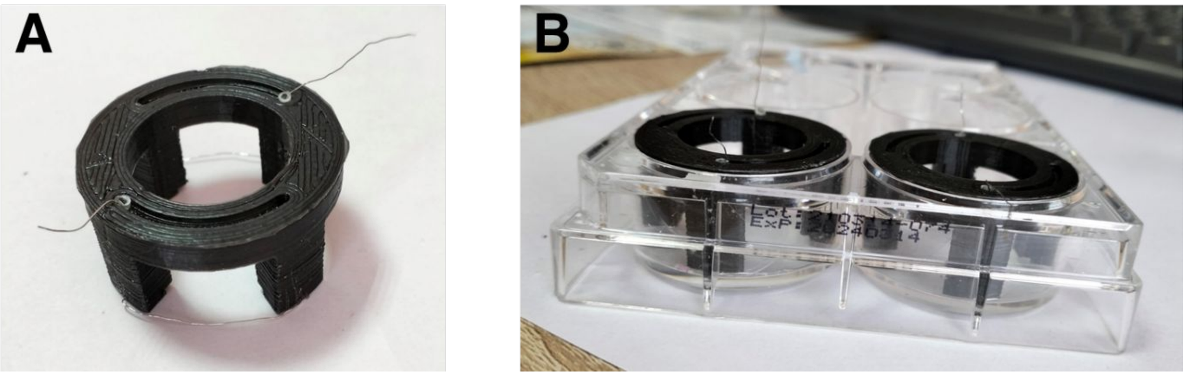

Supplementary Figure S11. The 3D printed inserts with platinum electrodes for the in-vitro electrophoresis. A, the insert with the electrodes; B, the inserts in the 6-well plate.

Supporting Information

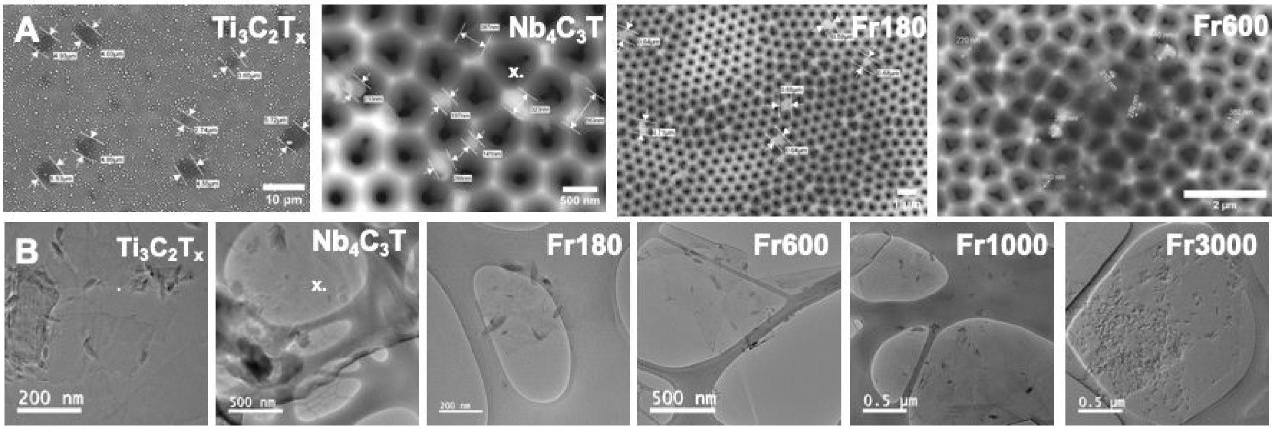

Supplementary Figure S12. (A) SEM and (B) TEM images of MXenes.

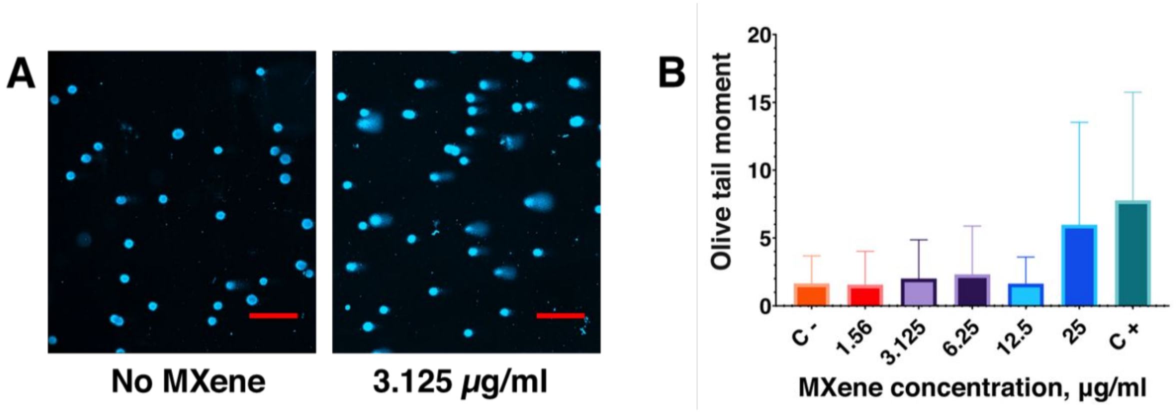

Supplementary Figure S13. MXene can induce comets at concentrations as low as 3 μg/ml. A, the B16F10 mouse melanoma cells were treated with $Nb_4C_3T_x$ MXene at 1.56, 3.125, 6.25, 12.5, and 25 μg/ml for 4 hrs, after which MXene was washed out, and cultivation continued for additional 24 hrs followed by the DNA comet assay; B, quantification of the resulting DNA comets. C+ stands for control cells treated with H_2O_2. Scale bars = 200 μm.

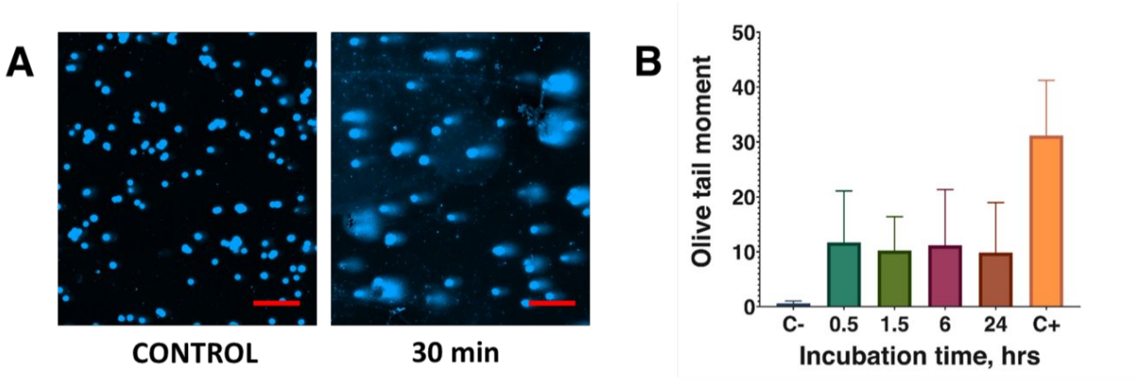

Supplementary Figure S14. MXenes are able to induce comets in living cells after 30 min of incubation. A, the B16F10 mouse melanoma cells were treated with 25 μg/ml of $Nb_4C_3T_x$ MXene for 30 min, 1.5 hr, 6 hr, and 24 hr, after which the cells were subjected to the DNA comet assay; B, quantification of the resulting DNA comets. C+ stands for control cells treated with H_2O_2. Scale bars = 200 μm.
